# Mechanistic insights and future perspectives of drought stress management in staple crops

**DOI:** 10.3389/fpls.2025.1547452

**Published:** 2025-03-27

**Authors:** Amir Abdullah Khan, Yong-Feng Wang, Rasheed Akbar, Wardah A. Alhoqail

**Affiliations:** ^1^ School of Environment and Safety Engineering, Jiangsu University, Zhenjiang, China; ^2^ Department of Entomology, Faculty of Physical and Applied Sciences, The University of Haripur, Haripur, Khyber Pakhtunkhwa, Pakistan; ^3^ Department of Biology, College of Science in Zulfi, Majmaah University, Al-Majmaah, Saudi Arabia

**Keywords:** drought stress, major staple crops, sustainable solutions, PGPB, hormones, nanoparticles, osmolytes

## Abstract

Due to extended periods of below-normal rainfall and rising temperatures, drought is a significant global issue for agricultural productivity. Hydrological, agricultural, and meteorological droughts all pose different problems with regard to the availability of water for important crops, which in turn impacts plant development and yield. Depending on the crop species and stage of maturity, drought stress degrades plant metabolism and physiological processes, resulting in decreased growth and yield losses that can range from 30% to 90%. Acclimatization and adaptation are the two basic techniques that plants use to survive drought. Rapid alterations in physiological processes and chemical composition, including modifications to osmotic pressure, root and leaf size, and antioxidant systems, are all part of acclimatization. Xerophytism and succulence are two characteristics that drought-resistant plants have evolved to assist preserve cellular integrity and water balance in water-limited environments. Even with these tactics, the majority of important crops—such as maize, rice, and wheat—remain extremely vulnerable to drought stress. To lessen the effects of drought, researchers have looked into a number of strategies, including both conventional and cutting-edge methods. Conventional techniques, like the application of plant growth-promoting bacteria (PGPB) and morphological modifications, remain essential for improving drought resilience. Recent breakthroughs have provided innovative alternatives such as nanoparticle (NP) treatments and biochar, which enhance plant resilience. Biochar enhances soil moisture retention and nutrient accessibility, whereas nanoparticles augment water absorption and bolster molecular resilience under stress. Furthermore, microbial inoculants such as plant growth-promoting bacteria (PGPB) enhance nutrient and water absorption, facilitating growth in arid conditions. This review examines the impacts of drought stress on three important staple crops, emphasizing both traditional and novel approaches to lessen the consequences of drought. We highlight how combining insights from ecology, biochemistry, molecular biology, and cutting-edge technologies like biochar and nanoparticles can boost agricultural production and plant resistance in water-scarce environments.

## Graphical Abstract

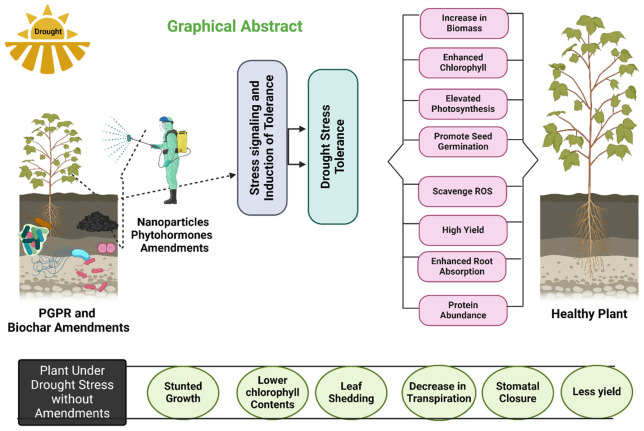

## 1 Introduction

Periodic droughts have a profound effect on the global food supply, particularly on staple crops such as rice, wheat, and maize, which are crucial for human nutrition and food security (Habib-Ur-Rahman et al., 2022). Drought is defined as a prolonged period of abnormally low precipitation that occurs at a specific location when the amount of rainfall consistently falls below the average. With respect to the world as a whole, agriculture is the main consumer of water, with almost 70% of all water taken from its sources. In underdeveloped nations, this number can increase to 95% (Kogan et al., 2019). Rain fed agriculture makes up a substantial majority of the world's farmed land, which is projected to be 1474 million hectares (Yohannes et al., 2024). Staple crops cultivated in these regions exhibit significant susceptibility to drought, characterized by a deficiency of water for plant growth. There are three primary categories of drought: hydrological, agricultural, and meteorological (Lima et al., 2019). Lower-than-normal precipitation, frequently coupled with warmer temperatures, can cause meteorological drought and other forms of drought. Reduced agricultural productivity results from agricultural drought, which is caused by either sporadic precipitation, excessive evaporation, or inadequate rainfall (Ali et al., 2021). A prolonged decrease in water availability that impacts lakes, reservoirs, aquifers, rivers, and streams is known as a hydrological drought. Droughts result from multiple factors, including inadequate precipitation, high temperatures, insufficient irrigation methods, and ecological concerns which include excessive grazing and erosion of soil. From the current situation to the 2090s, the forecasts show a considerable rise in the proportion of land surface area experiencing extreme drought. It is anticipated that the average duration of severe drought episodes would grow by a factor of six every century in the 2090s, and the frequency of these events will likely double (Burke et al., 2006). According to data from the World Bank, drought is a highly costly catastrophe that inflicts significant damage to the economic prosperity and food security of individuals who depend on agriculture. Agricultural scientists worldwide are working diligently to minimize crop water consumption, aiming to address the difficulties faced by farmers in drought-prone regions of the developing world. Given the limited availability of water resources, drought poses a significant and urgent challenge to global food security (Hasanuzzaman et al., 2013). It played a significant role in causing major famines in the past as well as the limited global water supply, we expect the growing demand for food due to population growth to exacerbate the impact of drought (Mullet, 2009). The severity of drought is intrinsically unknown because a variety of factors, including the frequency and distribution of rainfall, rates of evaporation, and soil moisture retention capacity, underscore the importance of drought resilience and sustainable agricultural measures practiced by agricultural scientists to ensure food security and high yields of staple crops. This review will help us understand 1) the specific impacts of drought on staple crops and global food security, 2) effect on overall plant health, and 3) the mechanisms by which these plants overcome drought stress and useful methods to address this challenge and ensure high yields.

### 1.1 Socioeconomic impact and recent catastrophic impact of drought

In addition to crop production, drought has significant and wide-ranging socioeconomic effects on rural livelihoods, food security, and economic stability (García-León et al., 2021). Water shortages, decreased agricultural output, and financial loss for farmers are all consequences of droughts, which are growing more common and severe as a result of climate change, particularly in developing nations (Lee et al., 2022). With repercussions on international food prices and trade, these effects worsen poverty, hunger, and inequality. In regions with limited water resources, the agricultural industry faces considerable challenges since drought stress hinders crop development and interrupts food production (Saha et al., 2023). Understanding the mechanisms underlying drought tolerance and creating robust crops will be essential to adjusting to future climatic scenarios, making research on drought stress imperative. To lessen the socioeconomic effects of drought and protect global food security in an increasingly uncertain environment, research investments in climate-resilient farming methods, enhanced water management techniques, and drought-tolerant crops are crucial (Patel et al., 2021). Climate change, inadequate water management, political unpredictability, and socioeconomic weaknesses have all contributed to the disastrous recent droughts in places like South Asia and Sub-Saharan Africa (Leal Filho et al., 2022). The most prominent of them is the devastating drought that struck Somalia (and the larger Horn of Africa) in 2016–2017, affecting millions of people and resulting in widespread displacement and food insecurity (Oğultürk, 2021; Awange, 2022; Dirie et al., 2024). In a similar vein, the periodic droughts in Afghanistan, Pakistan, and India are getting worse, severely affecting public health, agriculture, and water supplies. According to climate models, the frequency and severity of these catastrophic occurrences may rise (Alasow et al., 2024).

## 2 Deciphering crops responses to drought

Limited access to water quickly results in a shortage of water within the plant system. This process impacts the metabolism, growth, and physical characteristics of crops (Dirie et al., 2024). Dehydration causes severe changes in physiological processes, resulting in a decrease or complete halt in growth and posing a risk to the stability of crop production (Anjum et al., 2011). The decrease in agricultural yield due to drought in the field usually ranges from 30% to 90%, and this variation is dependent on the specific crop species (Hussain et al., 2019a). The effect of water scarcity on crop yield varies depending on the exact plant component being harvested, such as leaves, shoots, seeds, fruits, or tap roots. Droughts during various stages of crop growth can have a significant effect on crop yield (**Table 1**). To understand plant reactions to stress, it is crucial to differentiate between acclimation and adaptation, just as with any other form of stress (Wang H. et al., 2022). Acclimation occurs when a species changes the way its genes are expressed and how it develops within its own genomic repertoire to improve its growth and performance in response to changes in its environment, such as drought (Krasensky and Jonak, 2012). However, this acclimation maybe different in species depending on the temperate and tropical crops. Due mainly to variations in their environmental needs and evolutionary adaptations, temperate and tropical crops react differently to drought (Kumari et al., 2021). Wheat and maize are examples of temperate crops that are suited to areas with seasonal rainfall (Nguyen et al., 2023). They are especially vulnerable to dryness during crucial growth phases like flowering and grain filling. In order to preserve moisture, these crops frequently close their stomata in response to water stress; nevertheless, if drought strikes during the reproductive stages, their output may be severely reduced (Krasensky and Jonak, 2012). Conversely, growing in warmer, more reliably wet climes, tropical crops like rice and soybeans are equally susceptible to drought, particularly during blooming and pod formation. Although tropical crops usually have defenses against brief dry spells, they are extremely vulnerable to extended water scarcity, which can negatively impact the development of grains or seeds. Rice and other tropical crops depend on constant water supply, whereas soybeans modify their root systems to reach deeper moisture. As climate unpredictability increases, both crop types must adapt drought-resilience techniques to suit their respective environments (Kunert et al., 2016).

**Table 1:** Yield reduction of crops under drought stress.

Crops	Growth stage	Yield reduction	References
*Zea mays* L.	Vegetative, reproductive, grain filling	25-92%	(Monneveux et al., 2005; Bonea, 2020)
*Oryza sativa* L.	Reproductive and grain filling	30-92%	(El-Lattef et al., 2011; Kumar et al., 2021; Ghouri et al., 2022)
*Zea mays* L.	Reproductive and vegetative	17-32%	(Hunter et al., 2021)
*Triticum aestivum* L.	Reproductive	20-25%	(Saeidnia et al., 2023)
*Triticum aestivum* L.	Grain yield	48-72.79%	Gudi et al., 2024
*Triticum aestivum* L.	Yield	25–29%	Sehgal et al., 2024
*Oryza sativa* L. *r*	Reproductive	53.7 to 63.5%	Jarin et al., 2024

### 2.1 Drought impact on nutritional value of staple crops

Cereals are essential in the human diet and have a crucial impact on global nutritional systems (Hassan et al., 2021). Wheat is the most important cereal crop for the continuously increasing global population, serving as the primary food supply for almost 40% of the world's population (Petronaitis et al., 2021; Wang Y. F. et al., 2022). Other key cereal crops, such rice and maize, are also grown and consumed globally (Soto-Gómez and Pérez-Rodríguez, 2022). A balanced diet is considerably aided by the abundance of proteins, carbs, and essential vitamins and minerals found in these three popular cereals. Wheat provides substantial levels of protein, including gluten, which is essential for baking and food processing, as well as complex carbs, which give us sustained energy. It additionally offers vitamins (including thiamin, niacin, and riboflavin), nutritious fiber, and essential minerals like iron and zinc (Saini et al., 2021; Kumar et al., 2023). In addition to being a staple food for over 50% of people worldwide, especially in Asia, rice is a major source of carbs (Bin Rahman and Zhang, 2023). It also acts as a priceless store of vital minerals including phosphorus, magnesium, and folate. Different varieties of rice, such as brown rice, provide additional minerals and dietary fiber compared to refined white rice (Longvah et al., 2021). Globally, maize is another important cereal crop that provides a number of vital elements and carbs (Poole et al., 2021). It contains vital elements such as niacin, vitamin B6, folate, magnesium, and phosphorus. Carotenoids and other bioactive compounds found in maize have antioxidant properties that are beneficial to human health (Kaushal et al., 2023). In addition to providing the required number of calories, these cereals enhance the overall nutritional value by providing a variety of essential elements for human health. They are crucial to food security and nutrition, especially in developing countries where they are the primary source of essential nutrients (Tanumihardjo et al., 2020). Developments in crop breeding, biotechnology, and farming practices are steadily enhancing the nutritional qualities and resilience of these grains, solidifying their vital role in the global food chain. However, the productivity and nutritional quality of these important grain crops are seriously threatened by drought stress (Tanumihardjo et al., 2020). For instance, wheat is adversely affected by drought stress, which lowers photosynthetic efficiency, inhibits root formation, and lowers germination rates. As a result, grain yields and biomass output are negatively affected (Vijayaraghavareddy et al., 2022). Plants' ability to absorb and transfer nutrients can be hampered by physiological stress brought on by drought. This may result in grains with lower amounts of essential minerals and protein (Zahra et al., 2021). Rice is far more vulnerable to drought circumstances than wheat since it is grown in paddy fields that require a lot of water. A lack of water for rice can lead to poor grain quality, reduced tillering, and insufficient grain filling. Moreover, drought stress reduces overall production and raises spikelet sterility. In regions with unpredictable rainfall patterns and water scarcity, rice's vulnerability to water availability is a serious concern (Ding et al., 2020; Arouna et al., 2023). Human health and food security are significantly impacted when crops under drought stress lose nutritional value. Crop production and nutritional density, particularly vital micronutrients like zinc, iron, and vitamins, are reduced during drought stress, which is typified by insufficient water availability. Studies reveal that extended drought circumstances can lower these nutrients' concentration in rice, maize, and wheat, among other staple crops (Zahra et al., 2021). In areas where staple crops are a major source of nutrition, this deterioration in nutrient quality increases the likelihood of micronutrient deficiencies, which can lead to a variety of health problems, including anemia, delayed cognitive development, and heightened vulnerability to infections (Zhang et al., 2018). Drought-induced nutritional shortages can worsen the incidence of chronic diseases, including cardiovascular ailments and metabolic syndrome (Kuromori et al., 2022). As drought stress escalates due to climate change, the transition to nutrient-deficient diets—typically consisting of drought-resistant, yet nutrient-poor, crops—exacerbates food insecurity. Furthermore, studies show the financial consequences for smallholder farmers, who suffer from decreased crop yield and quality due to drought stress, which lowers their income and makes food insecurity worse (Zhang et al., 2018). Sustainable food systems depend on techniques that increase crop nutritional value and drought tolerance in order to lessen these effects.

Grain filling and blossoming are two critical growth stages in maize that are significantly impacted by drought. Drought stress during these times can lead to diminished grain size and quantity, poor pollination, and kernel abortion (Wang et al., 2020a). Reduced water availability brought on by drought has a detrimental effect on photosynthesis and metabolic processes, which lowers plant growth and yield potential (Cai et al., 2020; Lobell et al., 2020).

The process of evolution leads to adaptation, which includes changes in genomic potential, such as in ecotypes, genetic variations, or extremophiles. Common adaption mechanisms that help organisms withstand dehydration include xerophytic traits and succulence (Geilfus and Geilfus, 2019; Laxa et al., 2019; Xu et al., 2019; Kuromori et al., 2022; Lim et al., 2022). Analyzing the connections between plant structure, function, and the environment at many stages of plant growth—including the organismal, cellular, and molecular levels—is essential to gaining a thorough grasp of adaptation (Barnabás et al., 2008). This review provides a concise overview of the following topics: (i) the influence of drought stress on cereal plant structure, chemical reactions, and molecular mechanisms; (ii) the consequences of water stress on crop yield; and (iii) the approaches used by staple crop plants to adapt to water scarcity, including improvements in drought tolerance within species. This review focuses on the biochemical, microbe-based, biochar, nanoparticle and other techniques that are used to overcome the issue of drought in plants.

### 2.2 Impact on developmental stages of crops

**Figure 1:**
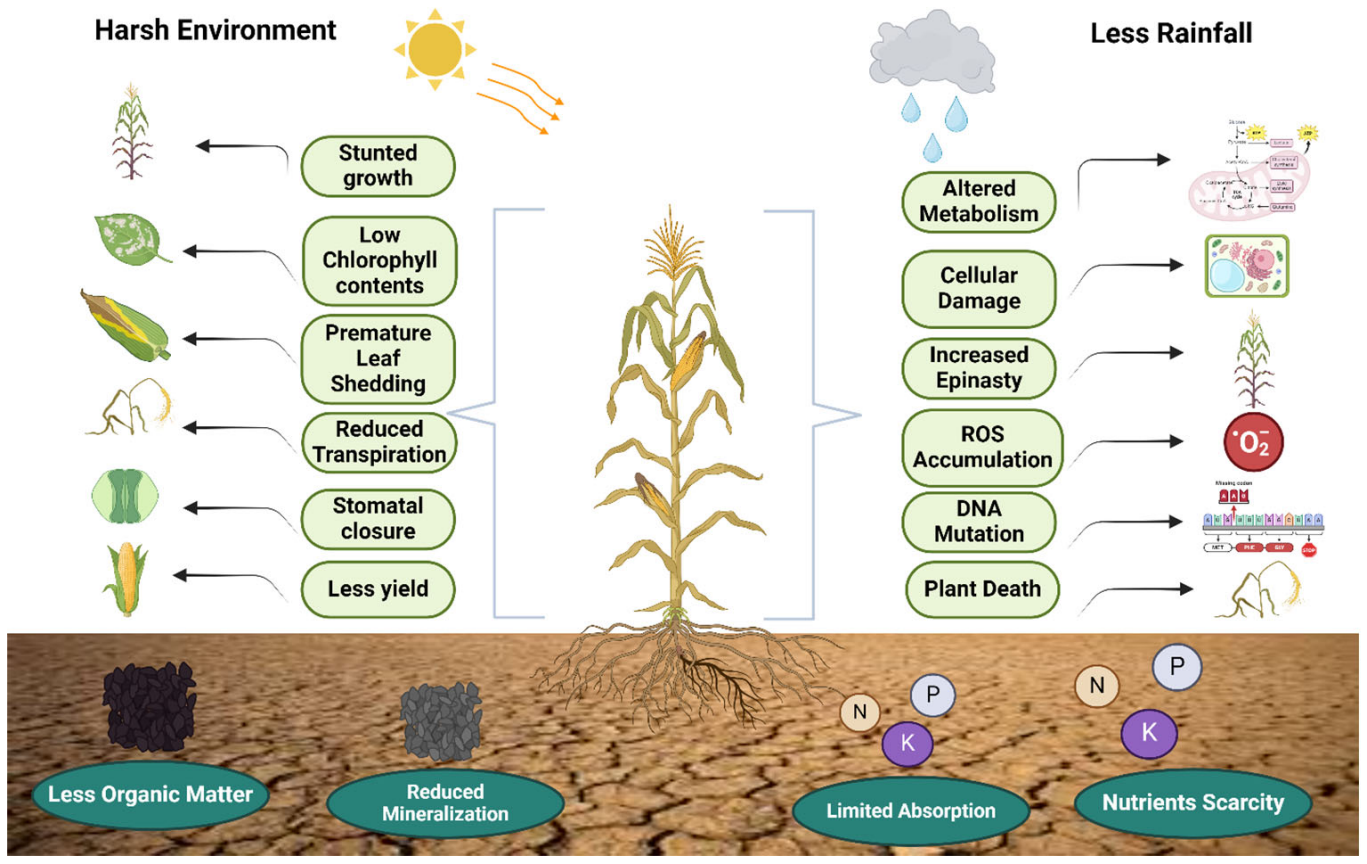
Negative effects of drought stress on the morphological, biochemical and environmental attributes of staple crops as well as soil. Drought stress effect both the physicochemical attributes of plants and soil which leads to decrease in growth and yield.

To ensure dependable crop yield, it is essential to consider both the duration of soil water scarcity and the particular phase of the plant growth cycle during a drought. Insufficient water during the growth stage reduces both plant growth and fruit or seed production (Farooq et al., 2009a), whereas water shortages during germination and seedling establishment can delay or inhibit germination, affecting the growth of seedlings and the process of photosynthesis (**Figure 1**). Focusing on the physiological and environmental effects, **Figure 1** graphically depicts the complex effects of drought stress on plants. Key elements that ultimately result in lower yields and nutrient scarcity are highlighted, including decreased growth, low chlorophyll content, and changed water and nutrient dynamics. This diminishes the vitality of plants. Yield represents the intricate synthesis of various growth stages. The impact of drought on yield is primarily contingent upon the plant's sensitivity to drought at various growth stages (Farooq et al., 2014). Drought during the vegetative phase resulted in a yield reduction of 21–50.6% for rice, while severe drought during the flowering stage led to a decrease of 42–83.7%. Additionally, moderate to severe drought throughout the entire reproductive stage caused a yield reduction of 51–90.6% (Zhang et al., 2018). A meta-analysis revealed that rice is more vulnerable to drought during reproductive stages like blooming, filling, and maturity, as opposed to vegetative stages like tillering and jointing. Similar findings in other research work have established that significant yield reduction transpires during the reproductive stage (Daryanto et al., 2017), as crops exhibit limited recovery from drought-induced damage. During the vegetative phase, drought limited the production of carbohydrates, which affected cell division and growth by closing stomata and partially stopping photosynthesis. This was deemed reparable (Barnabás et al., 2008). Sarvestani et al. (2008) demonstrated that a water deficit during the flowering stage resulted in a 50% reduction in rice yield compared to deficits occurring during the vegetative and grain filling stages. These findings also align with those of Boonjung et al (Boonjung and Fukai, 1996), which indicated a rice yield reduction of approximately 30% attributed to decreased number of grains per panicle. The drought transpired during the vegetative stage.

## 3 Drought induced vulnerabilities in staple crops

Staple crops such as wheat, rice, and maize play important roles in ensuring global food security by supplying the majority of calories and nutrients for a considerable proportion of the world's population. Nevertheless, these crops are becoming more susceptible to drought, a significant abiotic stress that jeopardizes agricultural productivity. Plants respond differently to drought on the basis of the specific type of stress and its length, intensity, and stage of growth, which occur at different levels of organization (Sallam et al., 2019). To make crops stress tolerant, comprehending how plants react to drought is essential.

### 3.1 Morphological vulnerabilities

#### 3.1.1 Influence of water availability on seed germination

Water is an essential ecological element that impacts the processes of seed germination and seedling growth in cereal crops (Xue et al., 2021). Therefore, it has a crucial function in influencing the arrangement of plant life in space (Gan et al., 2021). Water is essential for seed germination, as it stimulates enzymes that start growth processes while helping in the breakdown of nutrients, permitting accessibility to the growing embryo. Appropriate humidity is needed to promote the process of dividing cells and elongation, which is necessary to guarantee the healthy and consistent germination of staple crops (Lesk et al., 2022; Lu et al., 2022; Reed et al., 2022). Drought stress has a profound effect on the developmental phases of plant growth (**Table 2**). However, seed germination is the most vulnerable period to environmental stress throughout the entire growth and development of a plant (Luan et al., 2021). Seed form, physiological structure, and genetic traits significantly influence seed germination. It is also intimately associated with environmental conditions, particularly soil moisture (**Figure 1**). The seed coat presents a range of colors as a result of diverse phases of seed maturation and other developmental variables (Kišš et al., 2021). Typically, seeds with dark coat colors have superior germination capacity compared with those with light coat colors, although there are exceptions to this generalization (Gao et al., 2023b).

**Table 2:** Effects of drought on various morphological features of major staple crops.

Crop/Plant	Morphological characters	Reference
*Oryza sativa* L.	Reduced plant growth and development at vegetative stage	(Ghouri et al., 2022)
*Oryza sativa* L.	Reduced plant growth and development at vegetative stage	(Ganie and Ahammed, 2021)
*Oryza sativa* L.	Stomatal closure, drying of leaf tip, root shortening	(Dash et al., 2018)
*Oryza sativa* L.	Reduced plant height, biomass and leaf area	(Mishra and Panda, 2017; Hussain et al., 2018)
*Zea mays* L.	Decrease in the height, diameter, dry weight, and fresh weight of the plant	(Hu et al., 2007; Naghavi et al., 2013; Balbaa et al., 2022)
*Zea mays* L.	Reduced plant growth and development at vegetative stage	(Lin et al., 2020)
*Zea mays* L.	Reduced stem length	(Ahmad et al., 2021a)
*Zea mays* L.	Root system alterations, stunted growth	(Hussain et al., 2020; Ranjan et al., 2022)
*Zea mays* L.	Delayed silking affecting grain yield, ear, and kernel number per plant	(Notununu et al., 2022)
*Triticum aestivum* L.	Root−shoot fresh and dry weights reduced; accelerate senescence	(Itam et al., 2020)
*Triticum aestivum* L.	Leaf rolling and curls	(Merrium et al., 2022; Yang et al., 2022)
*Triticum aestivum* L.	Effected ear and kernel development	(Zahra et al., 2021)
*Triticum aestivum* L.	Decreased number of grains per plant	(Haider et al., 2020)
*Triticum aestivum* L.	Decreased biomass and resulted in stunted growth	(Chowdhury et al., 2021)

The color of seeds, which may indicate their level of development, is obvious; therefore, color has been shown to be strongly correlated with water absorption (Yaman et al., 2020). The process of seed germination is also closely associated with the shape of seeds (Fernández-Pascual et al., 2021). Different studies have shown that smaller staple crop seeds facilitate the absorption of water more quickly through permeability than larger seeds do. The reason behind this is that smaller seeds possess thinner outer layers and comparatively larger areas, which increase their ability to absorb water (Ali et al., 2020a; Badr et al., 2020; Souza et al., 2021; Saeed et al., 2022; Albert et al., 2024). Another study on alfalfa seeds revealed that the color of the seeds plays a crucial role in easing the impact of drought stress. Similar experimental work has been conducted in staple crops such as wheat, maize, soybean, and rice. Seeds with a lighter color are more tolerant to drought stress (Khadka et al., 2020; Sun et al., 2022). An increase in drought stress significantly inhibits root length, hypocotyl length, and seedling fresh weight. Nevertheless, seeds of staple crops with lighter colors exhibit superior adaptability in dealing with drought-induced stress. Higher nutrient storage in light-colored seeds enhances their growth capacity (Khadka et al., 2020; Ma et al., 2021).

#### 3.1.2 Impact on crop growth phases and yield

**Figure 2:**
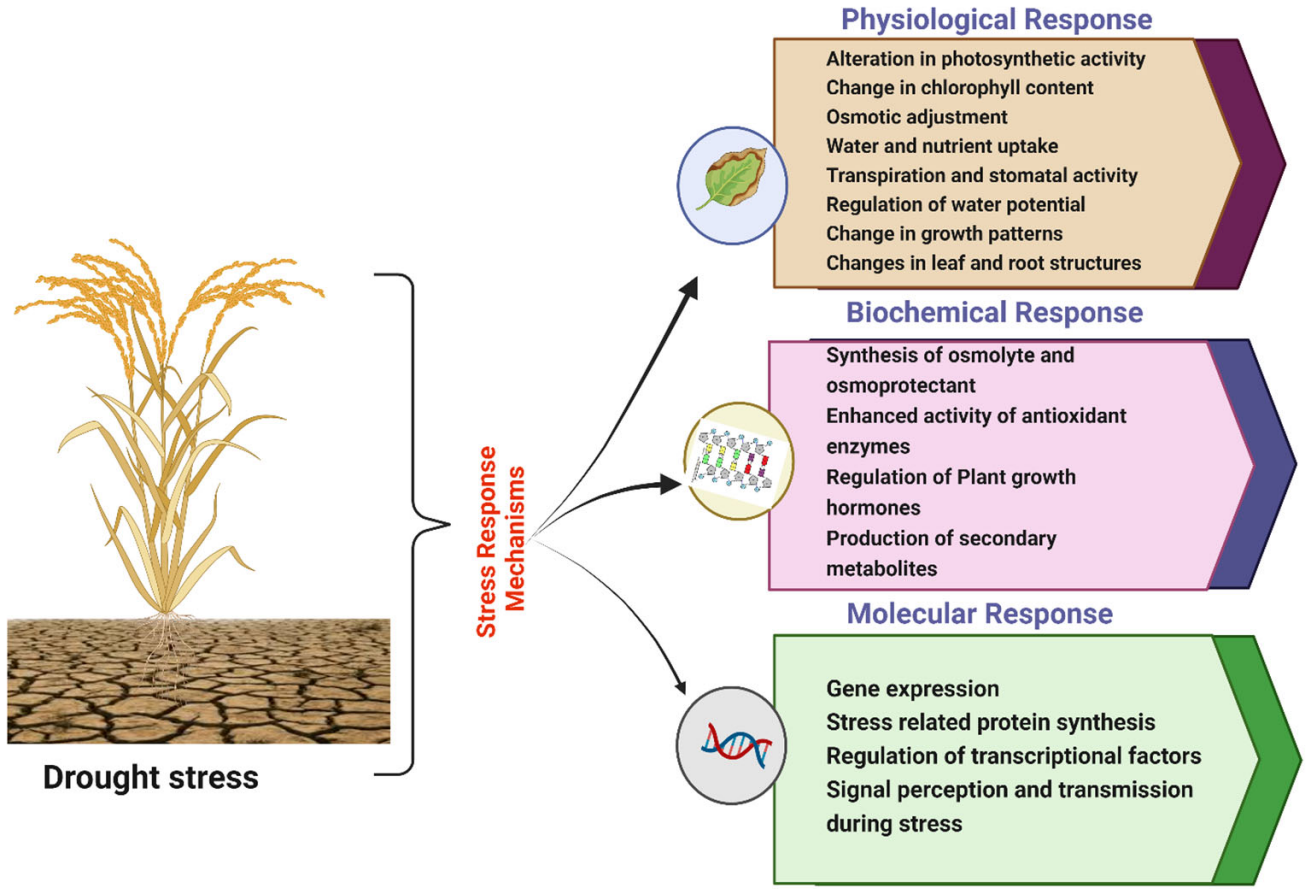
Different naturally built-in mechanisms in plants to overcome drought stress; physiological, biochemical and molecular responses. Plants have naturally building systems which help to prevent the plants from the negative effect of the drought.

Drought is a major contributor to global crop losses because it can reduce average yields by more than 50% (Anjum et al., 2017; Hussain et al., 2019b). The impact of water scarcity on crop productivity differs depending on the particular agricultural product under cultivation, including foliage, tubers, stems, fruits, or seeds. Furthermore, dryness during specific phases of crop growth can have substantial impacts on crop yield (Basu et al., 2016; Basu et al., 2022). Drought primarily reduces plant development by affecting cell division and expansion, which involves complex interactions of morphological, physiological, and genetic components that are affected mainly by water shortages, as shown in **Figure 2** (Wach and Skowron, 2022). The strategies that plant use to respond to drought stress are shown in **Figure 2**. It divides these reactions into physiological, biochemical, and molecular levels, highlighting adaptations including modified osmolyte production, photosynthesis, antioxidant enzyme activity, and gene expression that responds to stress. The intricacy of plant resilience is highlighted in this picture, along with areas that can be improved by breeding and biotechnology methods to increase drought tolerance. The growth of cells is a biological process that is most impacted by dryness, which primarily results from a decrease in the turgidity of the cells (Ozturk et al., 2021). Severe water scarcity can hinder the growth of staple crops and other plants by disrupting the flow of water from the xylem to neighboring elongating cells (Kasim et al., 2013; Dietz et al., 2021). Water stress causes disruptions in membrane electron transport, slower photosynthesis, damage from reactive oxygen species, reduced light absorption, and inefficient water use (Hasanuzzaman et al., 2013; Kumar et al., 2022).

Studies have shown that drought stress significantly impacts the germination of seeds as well as the establishment of wheat seedlings (Kasim et al., 2013; Mahpara et al., 2022). Research on drought stress in crops has mostly focused on reproductive development because drought during this period causes production to drop more significantly than at other phases, which has been discussed in relation to many staple crops like wheat, rice, and maize (Ji et al., 2012; Li et al., 2015; Senapati et al., 2019; Dietz et al., 2021; Yashavanthakumar et al., 2021). Other studies, however, have indicated that drought stress during the early vegetative stages or when seedlings are established might have a negative impact on crop development and output. Depending on the crop, the proportion of agricultural yield lost to water stress typically ranges from 30% to 90% (Hussain et al., 2019a). Drought conditions have a substantial effect on the yield of wheat, maize, rice and soybean seedlings or throughout their vegetative growth phases, leading to a decrease in production (Farooq et al., 2009b; Tabassam et al., 2014; Daryanto et al., 2016; Žalud et al., 2017; Dietz et al., 2021; Panda et al., 2021). Drought has been shown to reduce yields in a number of crop species; the extent of the reduction depends on the severity and duration of the stress period. **Table 1** provides a concise overview of numerous crops and plants, along with the impact of drought at various phases of growth and yield reduction.

#### 3.1.3 Reduction in leaf shape and size as key indicators of drought

Environmental factors, including the intensity, duration, and frequency of drought, soil characteristics, growth conditions, and plant species, have substantial effects on the severity and duration of drought-induced symptoms in plants (Egamberdieva et al., 2020). The primary markers of drought stress in plants are etiolation, wilting, loss of turgor, withered flowers, rapid senescence or abscission, brittleness or curling, burning and flaccidity, and leaf chlorosis (Rehschuh and Ruehr, 2022). Under drought conditions, plants may exhibit less common symptoms, such as twig cracks, stunted growth, branch dieback, necrosis, bark cracks, shrub canopies, and tree thinning (Toscano et al., 2021). Under certain circumstances, plants might perish due to severe drought-induced stress. Additionally, an abundance of water negatively affects plant function, impeding growth and diminishing the final output. Flaccids, succulent foliage, decayed foliage, and plant components impacted by fungal growth and mold are examples of flooding stress (Seleiman et al., 2021).

Many studies have shown that drought stress gradually reduces the size of leaves, leaf length, dry matter in shoots, and crop growth rate in wheat, rice and maize plants (Jurgens et al., 1978; Hu et al., 2006; Ahmad et al., 2018; Cai et al., 2020; Salgotra and Chauhan, 2023). Leaf area reduction is another morphological alteration in plants that triggers a cascade of interconnected activities, with photosynthesis being the most apparent outcome (**Figure 1**). The leaf area index (LAI) and leaf-specific activity are factors that contribute to photosynthetic performance in a field setting (De Costa and Shanmugathasan, 2002). Tarumingkeng and Coto (2003) researched the effects of drought on soybean plants and reported that a decrease in leaf area resulted in a reduction in the rates of photosynthesis and transpiration. Soybean plants with larger leaf areas have a greater capacity to provide more photosynthetic energy to the seeds. Water-stressed plants, however, experience a decrease in total photosynthesis due to reduced carbon fixation per unit leaf area caused by premature stomatal closure and nonstomatal inhibition of the photosynthetic machinery, as shown in wheat, maize and rice plants under drought (Arifuzzaman et al., 2020; Cai et al., 2020; Pour-Aboughadareh et al., 2020; Pitaloka et al., 2022). Additionally, there was a decrease in the photosynthetic surface area due to reduced leaf enlargement. Consequently, water-stressed staple plants exhibit simultaneous decreases in rapid leaf senescence, stomatal conductance, photosynthesis, and transpiration (Cai et al., 2020).

#### 3.1.4 Variation in root architecture in response to drought

Drought impacts all stages of plant development, including the germination of seeds, the establishment of seedlings, and the growth of shoots and roots. This covers the impact on the storage tissue of sugarcane, the roots of staple crops, the soft taproots of carrots and sugar beets, and the tubers of potatoes, among other specific plant structures (**Tables 1**, **2**). It has been demonstrated that roots can sense when there is not enough water in the soil and can then communicate to slow down the rate at which water vapor escapes through stomata and produce leaves (Schachtman and Goodger, 2008). Studies have been carried out to examine how drought affects crop or plant roots by quantifying root characteristics including length, surface area, or volume (Huang and Gao, 2000; Comas et al., 2013; Boguszewska-Mańkowska et al., 2020), elongation rate (Bengough et al., 2011), and spatial structure (Wang et al., 2011). The importance of comprehending how crop root systems is affected by water scarcity stress is underscored by these investigations (Kumar et al., 2018; Kumar et al., 2022). They frequently stated that crop roots react to water stress and that root properties are influenced by soil moisture levels (Chun et al., 2021). When maize plants are subjected to drought, architectural changes and a reduction in root size are noted (Messina et al., 2021; Ru et al., 2022). Other investigations have also documented similar responses from the roots of rice and wheat plants (Figueroa-Bustos et al., 2020; Li et al., 2021a) (Santos-Medellín et al., 2021; Gao et al., 2023a).

By analyzing variations in root biomass, researchers have investigated how drought stress affects the roots of staple crops (Chareesri et al., 2020; Gui et al., 2021; Li et al., 2021b; Hazman and Kabil, 2022), root length (Figueroa-Bustos et al., 2020; Tiwari et al., 2021; Bacher et al., 2022; Fonta et al., 2022), and diameter (Ahmad et al., 2021b; Kuhla et al., 2021; Li et al., 2021a; Li et al., 2021c). These findings indicated that the roots of these crops exposed to drought stress presented increased penetration into the soil, in contrast to the roots under well-watered conditions. Root elongation during drought can facilitate plant access to deeper water, thereby preventing water deficiencies in the upper layers of the soil. According to Ludlow and Muchow (1990), the development of roots could decrease water loss through drainage, as long as there is enough precipitation to allow for recovery after drought. Nevertheless, in cases where water is not easily reached in the lower layers of the soil, the presence of longer roots can lead to a reduction in the biomass of the aerial or plant parts above the ground and the harvest index. This occurs because the plant allocates more of its photosynthetic resources to the roots at the expense of shoot growth (**Figure 2**).

The root architecture significantly contributes to the effectiveness of water uptake and transportation in plants, thereby assisting in reducing damage resulting from drought stress. The root apex initially perceives drought-induced stress, detects the signal, and then conveys it to the plant's aerial portion (Jia and Zhang, 2008; Obidiegwu et al., 2015). In an effort to lessen the effects of water scarcity and unpredictable rainfall patterns, farmers are increasingly choosing drought-tolerant root crop varieties (Siddiqui et al., 2021; Maqbool et al., 2022). These cultivars, which were created by conventional breeding or genetic engineering, can increase water intake and decrease water loss to sustain consistent yields in arid environments. Farmers may improve food security, stabilize their incomes, and lessen their reliance on irrigation by utilizing these drought-tolerant crops, which will also increase the resilience of their farming systems to climate change (Nehe et al., 2021; Ober et al., 2021).Drought induces several alterations in the phenotypic attributes of roots, including changes in the total surface area, average diameter, total length, volume, and biomass of plant roots (Zhang and Sun, 2016; Zhang et al., 2019). Furthermore, the morphology and number of mitochondria and other cytoplasmic organelles are modified, particularly in the cells of the root (Liu et al., 2021). Under mild drought conditions, plants can increase their potential to withstand drought by elongating their primary roots and increasing the quantity of the lateral root system and root hairs (Salazar-Henao et al., 2016). However, during prolonged periods of dryness, the respiration of plant roots decreases, resulting in an inadequate provision of ATP. This leads to a substantial decrease in root activity, ultimately causing deceleration or complete cessation of growth (Kim et al., 2020).

### 3.2 Physiological vulnerabilities

#### 3.2.1 Drought alters the photosynthetic efficiency of crops

Photosynthesis is the primary physiological activity that is directly associated with the development, growth, and productivity of all photosynthetic plants (**Figure 1**). Chlorophyll, which is an important plant pigment, is produced in a cellular organelle, namely, chloroplasts, which are crucial for the process of photosynthesis. Chloroplasts use chemicals generated during photosynthesis and important proteins involved in metabolic pathways to ensure the tolerance of plants to abiotic stressors such as drought (Sun et al., 2009; Liu et al., 2020b). Chlorophyll, the primary constituent of chloroplasts, performs an important function in photosynthesis. However, oxidative stress, such as drought stress, negatively impacts chlorophyll synthesis in chloroplasts (Faisal et al., 2019). The decrease in chlorophyll content during drought stress is a result of pigment photooxidation and chlorophyll degradation (Nezhadahmadi et al., 2013). When plants are exposed to drought stress, it induces alterations in chlorophyll pigments (Anjum et al., 2011), disturbs the chloroplast apparatus (Fu and Huang, 2001), and halts the activities of enzymes responsible for the Calvin cycle, which results in lower yield production by crops (Monakhova and Chernyad'ev, 2002).

When the quantity of accessible soil water is moderately or severely restricted, plants respond by closing their stomata (Farooq et al., 2009a). This leads to a decrease in the amount of carbon dioxide entering the leaves, which in turn increases the number of electrons available for the generation of reactive oxygen species. This leads to decreased transpiration, resulting in increased heat retention inside the leaves (Yokota et al., 2002). Many research findings frequently link stomatal reactions to soil moisture levels rather than leaf water status (**Figure 1**). This implies that stomata respond to chemical cues such as abscisic acid (ABA), which are generated by depleting roots while maintaining a constant leaf water status (Turner et al., 2001).

Severe drought conditions limit the process of photosynthesis by decreasing Rubisco activity in different staple crop species (Chen et al., 2021; Luo et al., 2021; Todorova et al., 2022; Luo et al., 2021). In maize plants, the activity of the electron transport chain is precisely regulated by the CO_2_ content in the chloroplast and modifications to photosystem II during drought circumstances (Correia et al., 2021). Cells become smaller and lose volume when dehydrated, which raises the viscosity of the cellular material. Protein aggregation and denaturation can result via interactions between proteins, which are more likely to occur when viscosity is elevated. The activity of enzymes, especially those involved in photosynthetic processes, may be negatively impacted by increased cytoplasmic viscosity caused by a rise in solute concentration (Hoekstra et al., 2001; McDowell et al., 2022). Lower levels of Rubisco, the maximum rate at which Rubisco converts ribulose-1,5-bisphosphate, the rate at which ribulose-1,5-bisphosphate is regenerated, stromal fructose bisphosphatase activities, and the efficiency of photosystem II in higher plants all contribute to a decrease in photosynthesis during drought. Furthermore, Rubisco works more as an oxygenase than a carboxylase during times of severe drought because its capacity to perform carboxylation is greatly diminished (Yang et al., 2021).

Meanwhile, as several studies have shown, reduced leaf water potential during water scarcity affects the activities of other enzymes (Cai et al., 2020; Kuhla et al., 2021; Mahpara et al., 2022). These enzymes are fructose-1,6-bisphosphatase, pyruvate orthophosphate dikinase, phosphoenolpyruvate carboxylase, and nicotinamide adenine dinucleotide phosphatemalic enzyme. The activity of pyruvate orthophosphate dikinase is reduced by 9.1 times when water is scarce. Compared to the other enzymes, which showed a decline of two to four times, this is significantly larger. These leads imply that the enzyme most likely to limit photosynthesis under water stress is pyruvate orthophosphate dikinase (Farooq et al., 2009a). Evidence suggests that delayed ATP production by photophosphorylation is the main factor limiting photosynthesis, even in mild drought circumstances (Lawlor and Cornic, 2002).

#### 3.2.2 Implications of drought on respiration in crops

Drought frequently results in a decrease in respiration in several plant components, including roots, shoots, leaves, flowers, and entire plants (Hussain et al., 2019a, b). Despite the extensive understanding of the impact of drought on photosynthesis, research on its influence on respiration is scarce (**Figure 1**). In contrast to photosynthesis, respiration is a continuous process that mirrors the entire metabolic activity of a plant (Mazahery-Laghab et al., 2003). However, there is a notable discrepancy in the way that dryness affects plant respiration. Plants may sustain or even enhance their respiration rate in the face of insufficient water supply, according to several studies, including those conducted on drought-stricken wheat and maize (Fang et al., 2022; Xu et al., 2022). But even in cases of mild drought, other investigations have also documented a total cessation of respiration (Flexas et al., 2005; Rani et al., 2020).

#### 3.2.3 Relative water content

One crucial metabolic feature that affects plant water relationships, transpiration rates, stomatal resistance, and leaf water potential is the relative water content (RWC) (Guerfel et al., 2009). The RWC is an important regulator of metabolic activities in plant tissues and a measure of a plant's degree of hydration. Depletion of water due to transpiration and root absorption raises RWC (Georgii et al., 2017). Many physiological processes, including photosynthesis, stomatal motility, cell proliferation, and overall plant survival, depend heavily on the leaf water potential (Alghabari et al., 2015). Resistance to mild to moderate water scarcity is made possible by maintaining the leaf water potential. Nevertheless, the efficiency of photosynthesis is reduced as a result of the higher loss of leaf water potential brought on by increased water stress (Nikinmaa et al., 2013). Numerous investigations have revealed that wheat has a lower relative water content (RWC) (Badr and Brüggemann, 2020), similarly in maize (Badr and Brüggemann, 2020; Cai et al., 2020) and rice plants (Salsinha et al., 2020; Salsinha et al., 2021). Similar outcomes for tomato plants and caper bush (Capparis spinosa) have also been documented (Subramanian et al., 2006; Aytaç et al., 2024) and for soybean roots, leaves, and pods (Samarah et al., 2006). However, the response of different genotypes, on the other hand, differed. For example, when plants are drought tolerant, their leaf water potential remains greater for longer periods of time than when plants are sensitive to drought. The impacts of drought are contingent upon both duration and intensity. The water content of tissues decreases in a linear manner as the degree of drought increases (Farooq et al., 2012).

#### 3.2.4 Stomatal movement and transpiration

Placing emphasis on more intense stressors hinders a plant's ability to cope with simultaneous heat and drought stress (**Figure 1**). More precisely, in times of drought, plants close their stomata earlier than usual to reduce water loss. However, during periods of heat stress, stomatal conductance increases, helping to lower leaf temperature by transpiring water. Stomatal constriction is the main physiological reaction of plants to water deficiency. Stomatal constriction also prevents water loss from the cell and so ensures the cell turgidity and osmotic pressure (Sadok et al., 2021).

**Figure 3:**
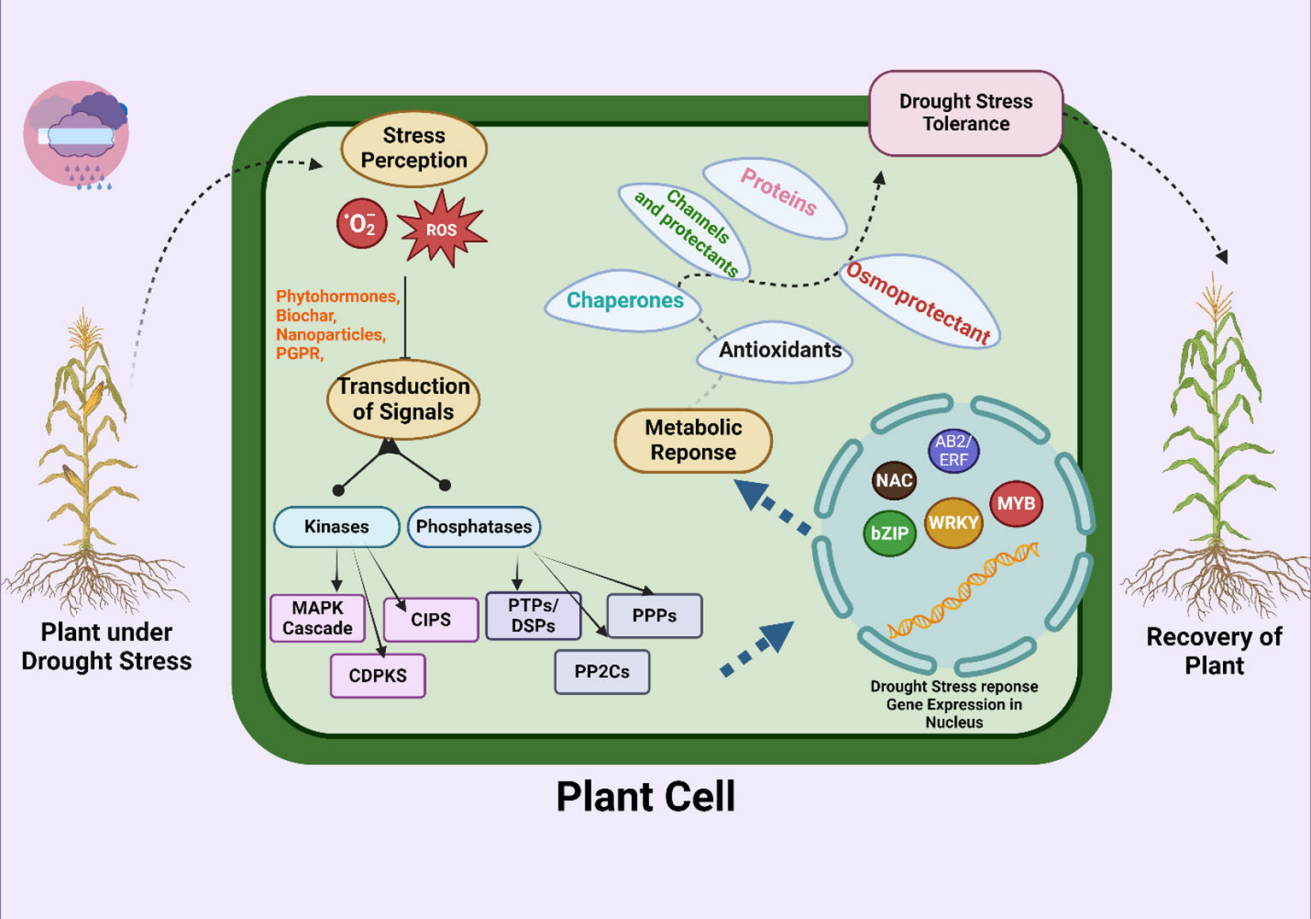
Molecular pathways adopted at the cellular level to overcome drought stress and their activation by different amendments. The signal transduction process involves various enzymes, including kinases and phosphatases. Important enzymes include the mitogen-activated protein kinase (MAPK) cascade, calcium-dependent protein kinases (CDPKs), protein tyrosine phosphatases/dual specificity phosphatases (PTPs/DSPs), phosphoprotein phosphatases (PPPs), and type 2C protein phosphatases (PP2Cs). These signal transmissions cause metabolic responses, such as the production of antioxidants, chaperones, osmoprotectants, and proteins, that increase the ability of plants to handle drought stress. Transcription factors, including NAC, bZIP, WRKY, MYB, and AB2/ERF, play a role in enhancing the ability of plants to recover from drought stress by regulating gene expression in the nucleus.

**Figure 4:**
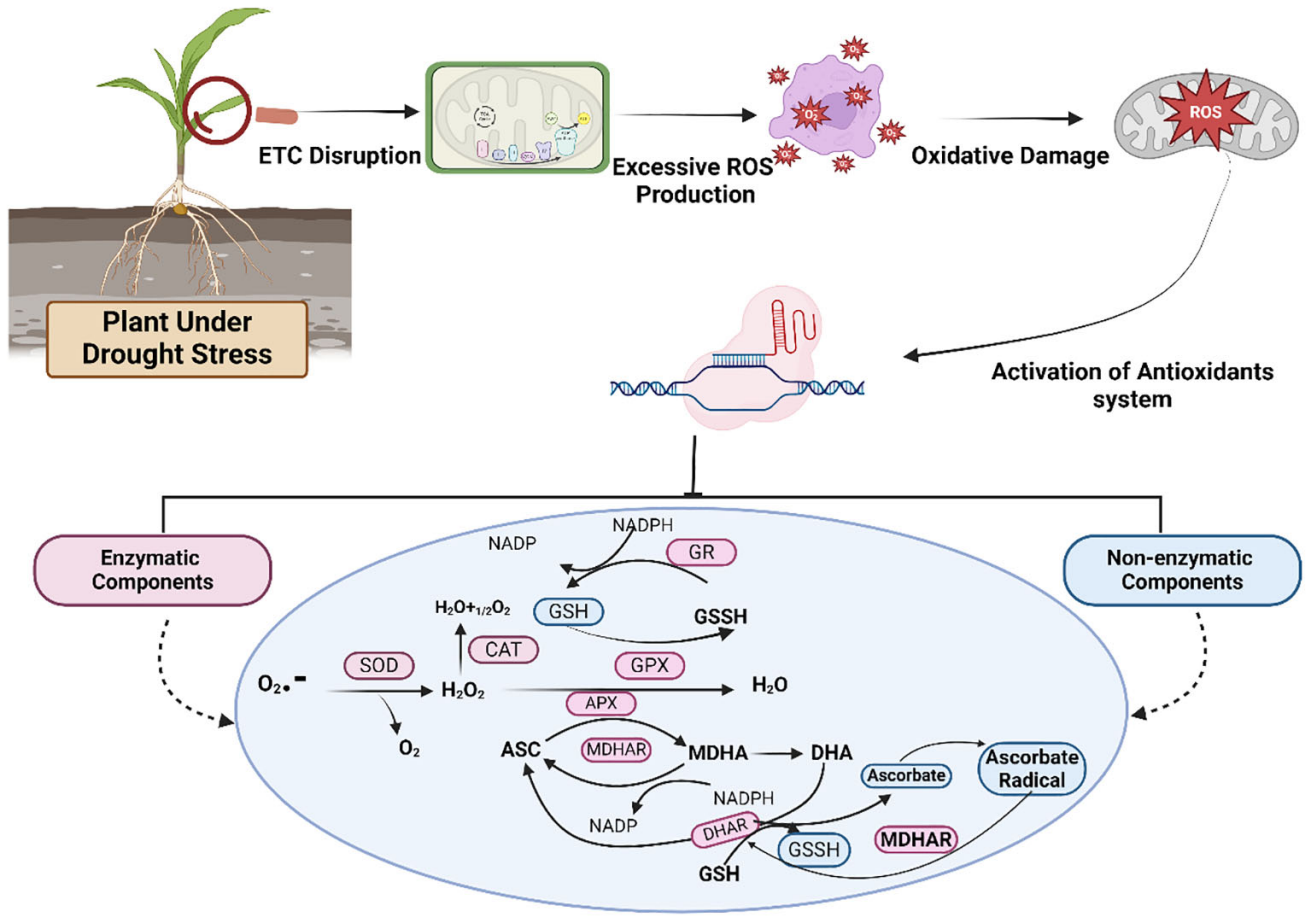
A detailed pathway and activation of enzymatic and nonenzymatic components of antioxidants to mitigate oxidative stress in staple crops under drought conditions. Different enzymes are involved in the pathway, including superoxide dismutase (SOD), catalase (CAT), ascorbate peroxidase (APX), glutathione peroxidase (GPX), glutathione reductase (GR), monodehydroascorbate reductase (MDHAR), dehydroascorbate reductase (DHAR), reduced glutathione (GSH), and glutathione disulfide (GSSH).

Water shortage conditions stimulate the synthesis of abscisic acid (ABA), which causes the stomata to close and triggers the activation of genes related to drought stress (**Table 3**; **Figure 3**). This helps regulate the plant's response to water scarcity (Bashir et al., 2021c). The presence of ABA in plant cells triggers ROS generation (Jiang and Zhang, 2002; Liu et al., 2022) H_2_O_2_, a significant reactive oxygen species (ROS), has an important part in multiple plant metabolic processes, stress reactions, and programmed cell death (apoptosis) (Apel and Hirt, 2004), This regulates the opening and closing of stomata. Both abscisic acid (ABA) and hydrogen peroxide (H_2_O_2_) are crucial factors in contributing to water scarcity (**Figure 4**). ABA also causes guard cells to make more H_2_O_2_ through NADPH oxidase. This H_2_O_2_ helps close stomata when ABA is present. Researchers have observed this process in the epidermis of wheat, rice and maize leaves under drought (Li et al., 2020a; Kong et al., 2021; Haverroth et al., 2023).

**Table 3:** Major phytohormones and their functions during drought stress in staple crop species.

Phytohormone	Function	Reference
Abscisic acid (ABA)	Seed maturation and seed dormancy in wheat and maize plants.	(Cheng et al., 2020; Bahrabadi et al., 2022)
	Regulate stomatal opening during drought in wheat, rice and maize.	(Du et al., 2018; An et al., 2019; Awan et al., 2021; Li et al., 2021)
	Trigger an array of biochemical defenses such as biosynthesis of proline, antioxidants, ROS detoxifying enzymes, heat shock proteins in wheat and rice	(Kaur and Asthir, 2020; Jiang et al., 2022)
Ethylene	Maintain Na^+/^K^+^ homeostasis and ROS production in maize.	(Ali et al., 2021a; Hussain et al., 2024)
	Reduce transpiration, constrict stomata, and thin the cuticle in wheat and rice.	(Iqbal et al., 2022)
Gibberellins	Seed germination, stem elongation, and reproductive development in maize crops.	(Wu et al., 2023b)
	Seed germination, internodal elongation, induced flowering, and fruit development in wheat and other staple crops.	(Islam et al., 2023; Sharma et al., 2024)
Auxins	Influence the plant growth responses during stress in crops.	(Tognetti et al., 2012)
	Involve in cell division, cell elongation, apical dominance, phyllotaxis, tropic responses, root branching in wheat and maize.	(Wang et al., 2020a; Todorova et al., 2022)
Cytokinins	Enhance photosynthetic activity in stress conditions in transgenic plants in wheat.	(Khosravi-Nejad et al., 2022)
	Cell differentiation, nutrient absorption, delayed senescence, flower and seed germination and development, and prevention of lateral root initiation in rice, maize and wheat plants.	(Gujjar et al., 2020; Liu et al., 2020a; Abdel Megeed et al., 2021)
Salicylic Acid	Enhances antioxidants system, water retention capacity and stomatal regulation in rice and wheat plants.	(Hosain et al., 2020; Shemi et al., 2021)
	CO_2_ assimilation, photosynthesis antioxidation, and stomatal regulation in wheat and rice plants.	(Munsif et al., 2022; Urmi et al., 2023)

#### 3.2.5 Oxidative stress

Oxygen metabolism naturally produces reactive oxygen species (ROS), which play a crucial role in cell signaling (**Figure 4**). However, in the presence of environmental pressures such as drought, there is a notable increase in reactive oxygen species (ROS) levels. This results in oxidative harm to proteins, lipids, and DNA, which in turn impairs normal cellular activities (Apel and Hirt, 2004). ROS such as O_2_^-^, OH-, and H_2_O_2_ directly attack lipid membranes and increase lipid peroxidation, resulting in an increased amount of malondialdehyde (MDA), which is responsible for cell membrane damage. Møller et al. (2007) established that MDA serves as a marker for oxidative damage and is the end result of the peroxidation process of unsaturated fatty acids in phospholipids.

The balance between the production of ROS and its breakdown regulates the concentration of ROS in cells in a steady state. Different environmental pressures, such as drought, disturb this equilibrium, leading to increased formation of reactive oxygen species (ROS) that exceed the capacity for scavenging, resulting in oxidative stress (Bashir et al., 2021c; Nayyar and Gupta, 2006), which leads to oxidative damage to the cell and eventually causes its death, as shown in **Figure 1** (Sharma et al., 2012). The inevitable flow of electrons onto oxygen from electron transport processes in mitochondria, chloroplasts, and plasma membranes continuously produces ROS. Numerous metabolic pathways in various parts of the cell can also generate ROS as byproducts (Sharma et al., 2012). As a result, it is critical to closely monitor and regulate the site and quantity of ROS production for signaling purposes, whether in response to stressful circumstances or during growth and development (Castro et al., 2021).

A sequence of interconnected events causes the synthesis of ROS during drought in different staple crops when they are exposed to drought conditions (Ahmad et al., 2018; Kumar et al., 2021; Ahmad et al., 2023). Initially, the closure of stomata hinders the transport of CO_2_ to Rubisco, leading to excessive accumulation of energy currency (ATP) and coenzyme (NADPH) in the Calvin cycle. Under such circumstances, dryness causes the photosynthetic electron transport chain to become saturated, especially when exposed to intense light. This results in an accumulation of surplus excitation energy in chloroplasts. Therefore, the reaction centers of photosystem I (PSI) and photosystem II (PSII) in the thylakoid membranes of chloroplasts produce reactive oxygen species (ROS) (Wach and Skowron, 2022).

### 3.3 Biochemical vulnerabilities

#### 3.3.1 Alteration in pigment content

Photosynthetic pigments, including chlorophyll, xanthophylls, and carotenoids, are essential for absorbing solar energy and converting solar energy to chemical energy, which involves carbon fixation. Chlorophyll is an essential element found in chloroplasts that drives the process of photosynthesis (**Figure 2**). The amount of chlorophyll present is directly related to the rate at which photosynthesis occurs. The marigold plants presented significant reductions in chlorophyll a and chlorophyll b levels due to drought stress (Asrar and Elhindi, 2011) and in the primary leaves of kidney beans (Miyashita et al., 2005). Drought has been shown to reduce the chlorophyll content in many plants, including cotton (Massacci et al., 2008), sunflower (Kiani et al., 2016), Catharanthus roseus (Jaleel et al., 2008), and *Vaccinium myrtillus* (Tahkokorpi et al., 2007). Shah et al. (2020), state that drought stress can significantly lower the amounts of chlorophyll a, chlorophyll b, and total chlorophyll in chickpea plants during their vegetative growth and blooming stages. Similarly, numerous studies have shown that when staple crops are subjected to drought stress, their photosynthetic pigments drop net (Moinuddin et al., 2005; Le et al., 2012; Kumar et al., 2018, 2021; Nasir and Toth, 2022). Photosynthesis depends on maintaining steady amounts of chlorophyll under drought because the plant uses chlorophyll to absorb light energy (Zahra et al., 2023). Chlorophyll can be weakened by water stress, which lowers a plant's capacity to generate energy. A plant that has stable chlorophyll can continue photosynthesizing, which promotes growth and drought resistance (Wang et al., 2021; Wang Y. F. et al., 2022).

One possible explanation for the decline in chlorophyll concentration in the leaves is the direct degradation of chlorophyll brought on by dryness (**Figure 1**). Under drought conditions, stomata closure is only one aspect of the complex process that limits photosynthesis. This phenomenon's main cause is usually a decrease in photosynthetic pigment concentration, which causes metabolic processes to be disrupted (Meena et al., 2021). Furthermore, as seen by decreased chlorophyll concentrations, dryness prevents plants from assimilating vital components, resulting in signs of element deficiency (**Figure 2**). Plant pigments change under drought stress, giving them yellow-brown tones. Studies reveal that plants with elevated levels of chlorophyll typically demonstrate robust drought resistance (Yang et al., 2021). All species capable of photosynthesis, as well as many organisms that cannot photosynthesize, produce carotenoids, a diverse group of isoprenoids, as shown in wheat under drought stress (Naderi et al., 2020; Simkin, 2021). Multiple studies have reported a decrease in carotenoid levels in different crops when they are exposed to drought conditions (Ali et al., 2020b; Nasrin et al., 2020; Javadipour et al., 2022; Simkin et al., 2022). They have a significant function as a defense system against oxidative damage, but they are very vulnerable to overactivation of ROS (Ashikhmin et al., 2023).

#### 3.3.2 Effects on nutrient uptake

Researchers have discovered that plants necessitate a minimum of 14 mineral elements, together with water, oxygen, and carbon dioxide, to obtain sufficient sustenance (Marschner and Rengel, 2023). The absence of these essential elements hinders plant growth and reduces crop productivity. Like other plants, staple crops also obtain these minerals from the liquid part of the soil. However, a lack of water due to dryness can impair the process of absorbing and moving nutrients, rendering them inaccessible for plant development and growth (Begna, 2021). Under drought stress, staple crops exhibit net decreases in the uptake of nutrients such as Na, K, Ca and Mg (Ostmeyer et al., 2020; Havrlentová et al., 2021). Potassium is a vital nutrient that enhances the endurance of plants in stressful situations (Khan et al., 2021a). When soil water levels decrease, plant access to potassium (K^+^) decreases, primarily due to the reduced mobility of K^+^ under stressful environmental conditions.

## 4 Intrinsic mechanisms by which plants overcome drought vulnerability

Drought management in plants is a multifaceted trait due to the involvement of several mechanisms. The primary plant approaches to drought include drought escape, drought avoidance, and drought tolerance. Plants undergo several physiological and biochemical changes when they are subjected to drought stress. Some of these changes involve changing how plants make food, making proteins that respond to drought, maintaining the balance of osmotic pressure, and activating antioxidant defense systems. When drought-induced stress is present, these systems collectively reflect the various levels of effect on plants **Figure 2** presents an overview of the response of plants to drought via various strategies.

### 4.1 Phenological adaptations as a key to drought tolerance

By using a mechanism known as "drought escape," plants are able to avoid exposure to drought conditions by completing their life cycle during times of abundant moisture. This frequently includes early flowering, accelerated plant development, quick germination, and seed production before dry spells (Ortiz et al., 2023). In order to avoid the worst times of water constraint, crops like wheat, rice, and maize adjust their development cycles using phenological strategies (Dietz et al., 2021). To successfully finish its life cycle before the onset of extreme dry circumstances, wheat may undergo early blooming and accelerated grain filling. Similar tactics may be used by rice, which is normally grown in regions with clear wet and dry seasons, by modifying its planting and maturation timetables to coincide with the availability of water (Moursi et al., 2021). In order to prevent drought, maize, which is particularly vulnerable to water stress during critical growth phases like blooming, can benefit from selecting cultivars with a shorter growing season or that mature early (Li et al., 2015). By aligning the most susceptible phases of plant development with times of more consistent water availability, these adaptation strategies lessen the detrimental impacts of water scarcity on agricultural yield. Through the synchronization of their development and reproductive periods with more favorable water availability, plants are able to avoid the negative consequences of drought.

### 4.2 Minimizing dehydration

Avoidance is another physiological approach adopted by plants under drought conditions (**Figure 2**). In general, plants avoid dehydration by increasing the thickness of the leaf cuticle, decreasing the leaf number and size and orientation, closing the stomata, reducing transpiration, increasing the photosynthetic capacity, improving water uptake, increasing root growth, and limiting vegetative growth (Hussain et al., 2019a). All these events help plants increase their water-use efficiency (WUE) and growth during periods of drought.

### 4.3 Drought endurance

Plant tolerance during drought refers to the ability of plants to endure and survive an extended duration of water scarcity, as mentioned in **Figure 2**. This involves various physiological, biochemical, and molecular adaptations that minimize water loss, sustain cellular activities, and protect against water stress (Moursi et al., 2021). These adaptations include altered stomatal density and closure, osmotic adjustment, the production of protective proteins, and the accumulation of compatible solutes to stabilize cellular structures, antioxidants, and ROS scavengers (Hasanuzzaman et al., 2013). Plant drought tolerance involves morphological, biochemical, and molecular changes. The display of one or more tolerance strategies determines a plant's ability to survive environmental stresses. An in-depth understanding of these systems can assist in choosing crop genotypes that are more resistant to drought. Plants possess several physiological, biochemical, and molecular pathways that increase their ability to endure dry conditions.

### 4.4 Physiological adaptations

Drought is a multifaceted stress that substantially affects the metabolism of plants and significantly constrains crop production (**Table 1**). The demand for irrigation water is continuously increasing while there is a significant decline in water availability; therefore, drought is becoming a major edaphic stress of future agriculture as well (Pandey et al., 2021). Plants can overcome water deficiency by reducing their leaf surface area, closing their stomata, or improving their water uptake by a root system that is deep in the soil (Niemczyk et al., 2023).

A crucial response of plants to water scarcity is growth arrest, as mentioned in **Figure 1** and **Table 1**. Water scarcity severely restricts the development of roots, shoots, and leaves, leading to stunted growth and development of plants. Restricted root development controls the function of the root meristem and promotes root growth when stress is alleviated (Xie et al., 2021). On the other hand, restricted shoot development reduces the metabolic requirements of the plant and concentrates the metabolites for the production of defense compounds important for osmotic adjustment.

The responses of plants to drought are multifaceted and vary greatly among different plant species, as well as across their developmental phases and the severity of water deprivation (Shahid et al., 2014). In addition to these changes, plants activate numerous biochemical processes ranging from controlled photosynthesis to antioxidant production and the accumulation of solutes, which are components of their resistance to water insufficiency. Plants respond to drought stress by regulating gene expression through intricate transcriptional networks (Singh et al., 2015).

### 4.5 Biochemical profiling

At the biochemical level, key molecules (carbohydrates, polyamines, and amino acids), plant hormones, and secondary metabolites contribute significantly to the stress tolerance mechanism (**Figure 2**). These molecules improve plant drought resistance by stimulating root growth, reducing leaf size and shedding, limiting ion leakage, removing ROS, maintaining proper osmotic pressure equilibrium, and stabilizing cell membranes (Sabagh et al., 2019). These mechanisms are described in detail below.

#### 4.5.1 Osmolyte and osmoprotectant biosynthesis

Osmotic regulation is considered an active process, and drought-stressed plants achieve osmotic regulation through three mechanisms: the accumulation of osmolytes, ion transport and compartmentalization, and water uptake and retention (Osakabe et al., 2014). Osmotic regulation maintains turgor pressure and stomatal conductance under water-deficient conditions and prevents the reduction of photosynthesis by maintaining high CO_2_ concentrations inside the mesophyll intercellular space, as shown in **Table 4**. It minimizes harm to the morphological, physiological, and biochemical pathways responsible for growth, photosynthesis, and stomatal conductance, as studied in different cereal crops under drought stress (Bashir et al., 2021b; He et al., 2021; Ullah et al., 2021; Yang et al., 2021).

**Table 4:** Important osmolytes and their functions during drought stress in three major staple crop species.

Osmolyte	Function	Reference
Proline	Enhance mitochondrial activity and regulate cell growth, thereby activating genes associated with drought-induced stress recovery in maize and wheat.	(Ibrahim et al., 2022; Zhang et al., 2022a; Li et al., 2024)
	Preserve the integrity of the cell membrane by reducing lipid oxidation, protecting the cell's redox potential, and decreasing the amount of ROS in rice.	(Hanif et al., 2021)
Glycine betaine	Improve the drought-resistant ability of maize plants.	(Shafiq et al., 2021)
	It enhances the plant's ability to regulate osmosis, control the opening and closing of stomata, and efficiently convert CO_2_ into carbohydrates, thereby facilitating the process of photosynthesis in wheat and maize.	(Shemi et al., 2021; Bai et al., 2022)
	Enhance the stability of the structure and characteristics of biological macromolecules in maize and other crops (enzymes of the dicarboxylic acid cycle, terminal oxidases, and photosystem)	(Dikilitas et al., 2020; Hossain et al., 2021)
Trehalose	Enhance photosynthetic performance by stabilizing macromolecules such as lipids, proteins, and other biological components in rice and maize plants.	(Lee et al., 2003; Joshi et al., 2020b; Zhang et al., 2022b; Mohanan et al., 2023)
Soluble sugar (glucose, sucrose, fructose)	Reduce the water potential of cells and improve plant capacity to absorb water and hold water in rice and maize plants. Maintain the protein structure and function by forming hydrogen bonds in maize.	(Gujjar et al., 2021; Xiao et al., 2024; Yao et al., 2024)

Plants regulate their osmotic balance by producing and storing osmolytes or osmoprotectants, which might be organic compounds or inorganic ions obtained from the surrounding environment. Osmolytes, or osmoprotectants, are essential for improving plant tolerance during periods of stress, such as drought and heat. Osmolytes are compatible, small molecules that are nontoxic and electrically neutral. They maintain the water-holding capacity of the cell during drought without hindering normal cell function (Wach and Skowron, 2022) and protect protein activity and cell membrane structure (Yang et al., 2021). Osmoprotectants are generally classified into three categories on the basis of their chemical nature. Sugars and sugar alcohols contain osmoprotectants (sorbitol fructan, mannitol, trehalose), amino acids containing osmoprotectants (proline, ectoine), and osmoprotectants containing ammonium compounds (glycine betaine, polyamines) (Blum, 2017). In different studies in staple crops the role of osmolytes have been documented showing their positive role in alleviation of drought stress and enhancing growth (Askari-Khorasgani et al., 2021; Ghosh et al., 2021; Ozturk et al., 2021; Pamungkas and Farid, 2022). For instance, Zhao et al. (2024) reported that wheat plants synthesize and accumulate PAs under drought stress, whereas Kubiś et al. (2014) reported that the external application of PAs provides protection against drought and enhances plant growth, photosynthesis, ROS scavenging, and osmotic regulation in wheat, maize (Anjum et al., 2017) and barley (Chen et al., 2019). The resistance of plants to drought is increased because of the increased accumulation of proline and glycine betaine (Ashraf and Foolad, 2007). In **Table 4**, the roles of important osmolytes are summarized. However, osmoregulation can only temporarily increase the drought tolerance of plants and has a limited impact. Plant turgor pressure cannot be maintained in the case of severe drought. Osmoregulation can only partially alleviate the damage to plants caused by water scarcity.

#### 4.5.2 Antioxidant enzyme production

Under drought stress, plants experience oxidative damage via excess production and accumulation of ROS (**Figure 4**). However, the extent, duration, and intensity of stress impact the severity of harm caused to plants. As previously mentioned, ROS have harmful effects on cell function by disrupting lipids, proteins, and nucleic acids (Hasanuzzaman et al., 2013). Stomatal closure decreases CO_2_ intake, making stressed plants more prone to oxidative damage. Under a normal water supply, there is an optimal equilibrium between the generation and removal of ROS, which might be disturbed via severe drought, resulting in high levels of ROS in plants. Thus, to reduce oxidative damage, plants enhance the synthesis of internal antioxidant defenses (Apel and Hirt, 2004; Møller et al., 2007; Kumar et al., 2018; Hussain et al., 2019b).

Plants activate an enzymatic or nonenzymatic antioxidant defense system to address abiotic stress, such as drought, as shown in **Figure 4**. Major enzymes include peroxidase (POD), superoxide dismutase (SOD), ascorbate peroxidase (APX), catalase (CAT), guaiacol peroxidase (GPX), ascorbate-glutathione (AsA-GSH), monodehydroascorbate reductase (MDHAR), glutathione reductase (GR) and dehydroascorbate reductase (DHAR) (Thabet and Alqudah, 2019; Wach and Skowron, 2022). They can either be directly involved in ROS scavenging or protect plant cells indirectly by activating a nonenzymatic defense system (Hancock et al., 2016). SOD is involved in the water−water cycle and the ascorbate−glutathione cycle across chloroplasts, mitochondria, cytosol, peroxisomes, and apoplasts. Conversely, CAT is present only in peroxisomes and is crucial for ROS scavenging (Mittler, 2002). Certain carotenoids and glutathione play essential roles as nonenzymatic components (Quan et al., 2016). Therefore, maintaining high levels of antioxidants might be an efficient way to control excessive ROS production in plants. In addition to their role as ROS scavengers and playing crucial roles in plant defense mechanisms, antioxidants also promote plant growth by regulating plant functions ranging from mitosis and cell elongation to senescence and cell death (Foyer and Noctor, 2005a; b). **Figure 4** briefly elaborates on various components of the antioxidant defense system.

These enzymes function within various subcellular compartments and synchronize their response when cells encounter oxidative stress. They either quench toxic compounds or regenerate antioxidants with the help of the reducing power provided by photosynthesis. Increased activities of antioxidant defense components have been reported in many studies of staple crops (Foyer and Noctor, 2005b; Alharby et al., 2021; Ali L. G. et al., 2021; Xu et al., 2022).

#### 4.5.3 Production of phytohormones

Plant hormones, also known as phytohormones, are vital biochemical compounds that significantly affect plant growth and development in different contexts, including during drought stress. They are crucial for plants to adapt to conditions of water scarcity (Raza et al., 2023). In addition to controlling stress responses, plant hormones also govern reactions to internal and external stimuli, as well as signal transduction pathways. A study on wheat revealed that phytohormones act as critical signaling molecules during the growth and development stages under drought (Abhinandan et al., 2018). Plant growth hormones are natural, organic, and small lipophilic compounds (Iqbal et al., 2023). Currently, nine types of phytohormones have been discovered, including auxins (the first phytohormone discovered), salicylic acid (SA), cytokinins (CKs), ethylene (ET), gibberellins (GAs), jasmonates (JA), brassinosteroids (BRs), abscisic acid (ABA), and strigolactones (SL), the most recently discovered phytohormones (Iqbal et al., 2022). Recently discovered as a phytohormone, melatonin plays a role in many physiological processes, including photosynthesis, growth, roots, seed germination, and defense against biotic and abiotic stressors (Arnao and Hernández-Ruiz, 2019). **Table 3** briefly explains important phytohormones and their role in plants. Plants require hormones at specific moments and locations during their growth and reproductive cycles, and they must nullify their effects when they are no longer necessary (Chumikina et al., 2019).

Low-molecular-weight PHs more frequently adopt the defense system of plants to respond to external stimuli accurately against stresses (Parveen et al., 2023). Owing to the chemical structures and physiological functions of phytohormones, only a few regulatory hormones, namely, Abscic acid (ABA), Jasmonic acid (JA), gibberellins (Gas), salicylic acid (SA), cytokinins (CKs), ethylene (ET), and Indole-3-acetic acid (IAA), have been studied by botanists (Li et al., 2020b).

Plants have evolved diverse morphological, physiological, and molecular strategies to regulate their cellular osmotic equilibrium (**Figure 2**). Therefore, research on these mechanisms is ongoing, as multiple PHs, which act as integral components of plants to manage and tolerate the negative effects of drought stress, are involved. The growth and development of plants are regulated by PHs, in addition to their drought stress effects throughout their lifespan (El Sabagh et al., 2022). When plants produce PHs under water deficit conditions, this triggers the pathway to manage their impact (Hamayun et al., 2021). Other developmental and physiological processes that PHs activate include stomatal closure, negative phototropism in roots, and osmotic balance (Lim et al., 2022).

Plant secondary metabolites are complex macromolecules that usually do not have a direct primary function (**Table 4**). Nevertheless, these chemicals mostly control plant maturation internally. Plants contain more than 100,000 secondary metabolites (Ahmad et al., 2023; Qiao et al., 2024). Secondary metabolites, in conjunction with diverse phytohormones, have functions in defense responses and signaling pathways. Their close linkage facilitates the development of efficient stress responses (Abu-Shahba et al., 2022). Drought is a multifaceted phenomenon that involves physiological, morphological, and biochemical alterations. The various functions of SMs include the regulation of enzymes, signaling, interspecies communication, and defense. SMs such as flavonoids, phenolics, flavonols, isoprenoid, isoprene, and phenylpropanoids act as antioxidants under drought stress (Nichols et al., 2015; Tattini et al., 2015; Naikoo et al., 2019). The accumulation of metabolites such as phenolics, flavonoids, diosgenin, glycosides, digitoxin, colchicines, glucosinolates, and saikosaponins occurs in response to stresses, which involve various elicitors and signaling molecules in different crops, including wheat and maize (Akladious and Mohamed, 2017; Dawood et al., 2022a, b; Ahmad et al., 2023). Moreover, polyphenols and flavonoids facilitate drought stress and allow plants to scavenge ROS during oxidative stress (Wahab et al., 2022). Diterpenes, other secondary metabolites, provide drought tolerance in wheat, maize and rice plants by inducing an ROS-scavenging system (Chatterjee and Pal, 2023). SMs act as antioxidants in plants to increase cell wall strength under stress (salinity, drought, heat, wounding, and herbivory) by reducing membrane lipid peroxidation and modulating the cell wall composition (Yang et al., 2021). Moreover, secondary metabolites enhance osmotic regulation in plants by improving glycolysis and the tricarboxylic acid (TCA) cycle to generate energy and facilitate the glutamic acid-mediated proline biosynthesis pathway (Quan et al., 2016).

#### 4.5.4 Molecular variations

Water scarcity has drastic effects on plant growth and development, as shown in **Figure 1**. This stress alters plant growth via alterations in various metabolic pathways, such as respiration, solute translocation, photosynthesis, mineral and ion uptake, transpiration, water potential, stomatal opening and closing, the antioxidant system, and phytohormone production (Seleiman et al., 2021). Under drought stress, plants shift their metabolism to activate numerous genes to mitigate the adverse impacts of stress (**Figure 3**), and the expression of these genes alters several biochemical and physiological systems (Anjum et al., 2011; Bashir et al., 2021c). The molecular mechanisms underlying drought tolerance can be divided into two main categories: i) Initially, transduction factors, which include ABA factors, protein kinases and transcription factors, are involved. ii) other factors include functional factors, the regulation of metabolic proteins, osmotic adjustment, the conversion of proteins to alleviate stress factors, protein conversion, protein modification, and the transport of reactive oxygen species (ROS) (Abhinandan et al., 2018).

When plants are stressed by water scarcity, the cell wall's breakdown first sets off stress-related signals by producing certain protein molecules (Levin, 2011). A number of proteins and metabolites, molecular chaperones, specific enzymes, and additional transcription factors (TFs) are among the signaling pathways that are triggered by drought stress (Scrimale et al., 2009). Different genes' expression under drought conditions was documented in earlier studies (Le et al., 2012). These drought-stress-related genes' activity indicates their participation in numerous cellular signaling pathways and reactions, including transcriptional regulation (Liu et al., 2020a). Transcription factors include Basic Helix-Loop-Helix (bHLH), MYC Proto-Oncogene (MYC), myeloblastosis viral oncogene homolog (MYB), NAM, ATAF1, and CUC1 (NAC), WRKY Domain (WRKY), Dehydration-Responsive Element-Binding (DREB), and Basic Leucine Zipper (bZIP), as well as protein kinases such as mitogen-activated protein kinases (MAPKs) and calcium-dependent protein kinases (CDPKs). They also include receptor proteins (Zhang et al., 2021). When wheat, maize, and rice plants experience water deprivation stress, they use both ABA-dependent and ABA-independent signaling pathways to detect and react (Shi et al., 2020; Zhao et al., 2020; Cao et al., 2021; Soma et al., 2021). ABA-independent transcription factors function as molecular switches in signal transmission. To directly regulate the expression of related genes, these factors interact with particular regions of the gene promoter called cis-elements (Zhao et al., 2021). The underlying properties of the DNA-binding sites are necessary for this inference. Certain genes' expression is regulated by transcription factor genes in response to drought stress (Long et al., 2020).

An important factor in stress signaling under stress is the increased release of ROS. Abscisic acid (ABA) activates ROS signaling, and plants under drought stress have elevated calcium levels (Abdel-Ghany et al., 2020). The excessive generation and accumulation of ROS in many plant tissues and cells are indicative of stress signals (Zhou et al., 2021). Moreover, the production of protective molecules, such as sugars, polyols, the amino acid proline (low-molecular-weight osmolytes), aquaporins, heat shock proteins, and late embryogenesis abundant proteins (LEA proteins), is involved in the response of plants to stress (Yu et al., 2020). These stress-related proteins protect cells from desiccation; regulate various biochemical, physiological, and morphological activities; protect the cell wall and integrity of the cell membrane; increase the biosynthesis of antioxidant enzymes to scavenge ROS; initiate or stop the physiological and biochemical processes involved in coping with the negative effects of stress; and stimulate water and ion uptake (Yan et al., 2020).

## 5 Human strategies to combat drought stress in staple crops

### 5.1 Culturing resistant plant varieties

**Figure 5:**
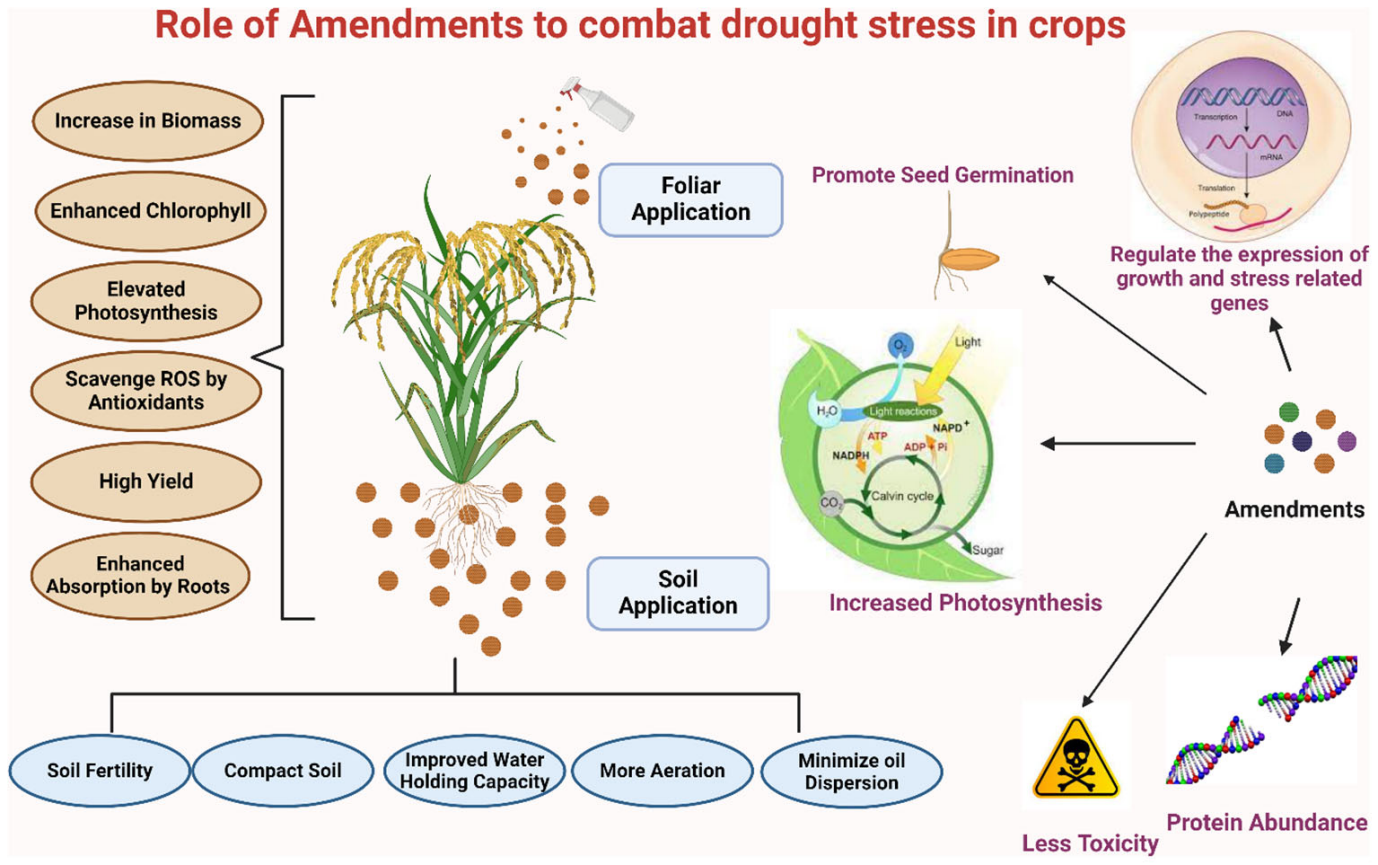
Strategic amendments to combat drought stress in staple crops: improving growth and tolerance.

To counter drought stress, the development of early-maturing crop varieties will make sensible use of the available soil moisture and result in relatively high yields. In plants, drought tolerance is controlled by additive and nonadditive gene effects under polygenic or oligogenic genetic control. Studying and characterizing drought tolerance is particularly difficult because of its complicated nature as a quantitative feature influenced by multiple genes (Maazou et al., 2021). Plants modulate various strategies to cope with or escape drought stress, either by growing excessively long roots to increase the uptake of water with a limited water supply or by reducing transpiration through leaves via stomata through the regulation of stomatal opening/closing (**Figures 2**, **5**).

Research has shown that treating wheat seedlings with substances such as calcium chloride (CaCl_2_) by osmopriming can effectively mitigate the adverse impacts of drought. The results of maize seedlings sprayed with CaCl_2_ unequivocally demonstrated enhanced drought resistance in both drought-tolerant and drought-susceptible cultivars (Abbas et al., 2021). Drought tolerance is a multifaceted strategy that encompasses physiological and metabolic pathways, as well as the genetic variety of plants (Mir et al., 2012). Biochemical, genetic, and physiological variables, as well as environmental impacts, influence routes in resistant varieties (Anderson et al., 2018). To better cope with current and expected future drought situations, drought-related crop research and practical implications need an exact and targeted approach. Molecular biologists’ cooperation with agronomists will help us understand the need for the day related to prevailing droughts and its drastic effects on food demand and security.

### 5.2 Applying nanoparticles in drought regulation

**Figure 6:**
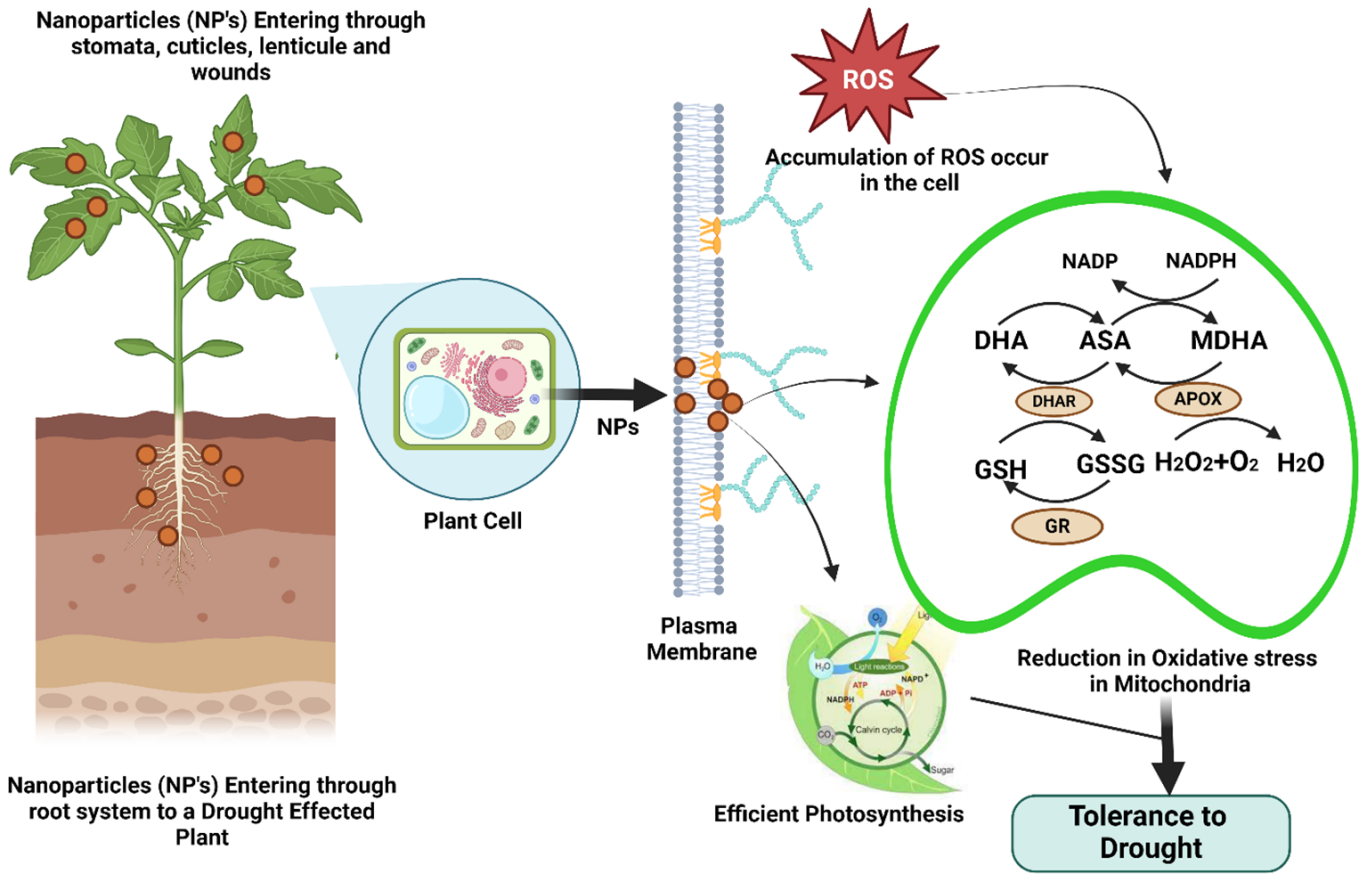
Nanoparticles (NPs) infiltrate plants through stomata, cuticles, lenticules, wounds, and the root system to staple crops under drought conditions. These NPs affect the generation of reactive oxygen species (ROS) within plant cells. To counteract the buildup of reactive oxygen species (ROS), the activation of antioxidant mechanisms is necessary. Key enzymes such as ascorbate peroxidase (APX), dehydroascorbate reductase (DHAR), glutathione reductase (GR), and monodehydroascorbate reductase (MDHA) play a part in these processes. This leads to a decrease in oxidative stress inside the mitochondria, facilitates effective photosynthesis, and improves drought tolerance.

NPs display increased reactivity because of their decreased size, expansive surface area, and meticulous structure (**Figure 6**). These substances selectively target plant cell organelles to release their contents in a regulated manner (Santana et al., 2020). Under stress conditions, nanoparticles play a significant role in the upregulation of antioxidant enzymes (CAT, SOD, POD, APX). Silicon-based nanoparticles have been reported to be effective at alleviating different abiotic stresses, i.e., drought, salt stress, heat, chilling, and heavy metal contamination (Tripathi et al., 2017). Mechanisms involved in the suppression of stress conditions include i) an increase in the antioxidant defense system in affected plants, ii) the compartmentation of metal ions, ii) coprecipitation, which refers to the association of substitute ions with harmful metal ions, and iii) immobilization, which refers to the confinement of toxic substances within the growth medium. A study conducted by Linh et al. (2020) revealed that metal-based nanoparticles, including copper, zinc oxide, iron, and cobalt, improve the ability of soybean plants to withstand drought stress. In another study conducted by Van Nguyen et al. (2022), copper-based NPs alleviated drought stress in maize plants by increasing antioxidant production. Similar findings were reported in rice plants subjected to drought stress when the seeds were primed with zinc oxide NPs (Waqas Mazhar et al., 2022). Silicon dioxide (SiO_2_) NPs subjected to drought conditions also presented a net increase in plant growth attributes and alleviation of antioxidants in response to the detrimental effects of drought (Sharf-Eldin et al., 2023). The application of NPs not only enhances the production of antioxidants but also increases the relative water content in stressed plants, reduces biomass loss, and improves the drought resistance index (Bashir et al., 2021a). Another study conducted by Sedghi et al. (2021) reported that the seed germination percentage and germination rates increase when plants are augmented with ZnO nanoparticles under drought stress. A recent study by Moolphuerk et al. (2022) reported that the application of chitosan nanoparticles enhances biochemical profiling and modulates the genes involved in the drought tolerance of rice plants. The use of nanoparticles offers creative solutions for energy, agriculture, medicine, and environmental sustainability, marking a substantial advancement in a number of disciplines. In these industries, their distinct physicochemical characteristics—such as their high surface area-to-volume ratio, adjustable reactivity, and capacity for tailored delivery—have created new opportunities for improving productivity and performance (Ashraf et al., 2021). However, a number of barriers prevent nanoparticles from being widely used, despite their potential (Wu et al., 2023a). The high cost of production is one of the main obstacles since creating nanoparticles frequently calls for costly raw ingredients, energy-intensive procedures, and specialized machinery (Fernandes et al., 2023). Because of this, they are less available, especially in areas with low incomes. Furthermore, there are substantial logistical and technical obstacles to overcome when transferring nanoparticle manufacturing from the lab to the industrial setting (Ying et al., 2022). There are still problems with preserving uniformity in size, shape, and purity as well as guaranteeing environmental safety throughout large-scale manufacturing. Commercialization is made more difficult by regulatory obstacles, which necessitate thorough safety evaluations due to worries about toxicity, environmental persistence, and bioaccumulation (Mazari et al., 2021). Limitations in infrastructure make it more difficult to deploy nanoparticle-based technology widely, especially in underdeveloped nations. Unlocking nanoparticles' full potential and guaranteeing their equitable and sustainable adoption across industries would require addressing these financial, technological, and regulatory obstacles (Jain et al., 2024).

### 5.3 Biochar amendment

Drought stress, along with the uptake of water and nutrients, also affects nutrient homeostasis, resulting in stunted growth and reduced yield. As a significant bioamendment, biochar improves water uptake and nutrient homeostasis, which leads to a substantial increase in the plant life cycle (**Figure 5**). Drought stress directly affects the process of photosynthesis by reducing the leaf surface area and disrupting the chlorophyll content and electron transport chain. But in order to boost plant development under drought stress, biochar amendment increases the synthesis of growth-regulating chemicals that enhance the metabolic pathways, chlorophyll content, and antioxidant enzyme activities (Manolikaki and Diamadopoulos, 2019; Lyu et al., 2022). Prior studies have shown that adding biochar lessens the negative effects of drought stress on the processes of carbon absorption and photosynthesis in important staple crop species like maize, rice, and wheat (Sattar et al., 2020; Hafez et al., 2021; Raza et al., 2021 (Anwar et al., 2023; Chowdhury et al., 2024; Kumar et al., 2024; Noureen et al., 2024; Shahzadi et al., 2024)). This is associated with a greater generation of chlorophyll and a less significant decline in the rate of stomatal conductance (Ran et al., 2020; Yang A. et al., 2020). Osmolytes play a vital role in counteracting the effects of drought stress (DS); however, studies suggest that DS disturbs hormonal equilibrium and the accumulation of osmolytes. Stress causes the production of proline, which is used for osmotic correction and as a scavenger of reactive oxygen species (ROS). Because drought stress raises ROS levels, it causes oxidative stress, which can harm vital plant components. Higher levels of superoxide dismutase (SOD) activity in water-stressed plants have been associated by researchers with enhanced defense against light damage and improved cell membrane integrity (Gharred et al., 2022). Researchers have shown that applying biochar to wheat boosts the expression of genes linked to drought stress (Mansour et al., 2019). Compared to plants subjected to 75% and 100% field capacity (FC), plants exposed to 50% FC have higher levels of expression of the CAT, APX, and Mn-SOD genes (Wu et al., 2023b). Even so, adding both BC and vermicompost led to lower levels of expression for the CAT, APX, and Mn-SOD genes, regardless of the amount of water added (Hafez et al., 2021). Drought stress hinders plant growth by reducing photosynthesis and nutrient absorption and generating ROS. Nevertheless, the application of BC improves plant nutrition, antioxidant activity, and the accumulation of osmolytes, resulting in increased growth and biomass output, has been documented in many studies of staple crops (Sattar et al., 2020; Bashir et al., 2021a; Gharred et al., 2022; Zulfiqar et al., 2022; Nawaz et al., 2023).

### 5.4 Utilizing PGPB to mediate drought tolerance

Plant growth-promoting bacteria play important roles in microbial communities linked to plants in a variety of situations (**Figure 5**). These bacteria rely on organic chemicals found in plant rhizospheres to sustain themselves and promote the growth of the plant. Plant growth-promoting bacteria (PGPB) enhance plant growth if the interior of the root surface or rhizoplane is an intracellular PGPB (Yadav et al., 2020), whereas extracellular PGPB reside on the surface of the root or the cortex region and colonize the intercellular spaces of the tissues (Glick and Glick, 2015; Abdelaal et al., 2021). In 1926, scientists first recognized the phenomenon of endophytic growth as an advanced phase of the bacterial life cycle (Mano and Morisaki, 2008; Fadiji and Babalola, 2020; Wang et al., 2021). Several endophytes can be isolated from surface-disinfected plant tissues (Mondal et al., 2022). *Bacillus, Azospirillum, Micrococcus, Pseudomonas, Azotobacter, Kocurria, Erwinia*, and *Serratia* are the genera of extracellular PGPB, whereas Rhizobia, which comprises *Bradyrhizobium, Mesorhizobium, Allorhizobium*, and Frankia, are among the intercellular PGPB (Khan et al., 2021b; Sagar et al., 2022). Growth-promoting microbes directly increase plant growth by increasing phytohormone synthesis and increasing the availability of nitrogen, iron, and phosphorus nutrients (Rostamian et al., 2023). Indirect antagonistic effects induce tolerance against phytopathogens (Glick and Glick, 2015). The strategies used by plants to promote growth under normal and stressed conditions include the secretion of phytohormones and siderophores; nitrogen fixation; phosphate solubilization (Singh et al., 2015); and the upregulation of defense-related enzymes, including IAA, abscisic acid, chitinase, ethylene, and gibberellic acid, which maintain the root system according to stress conditions that lead to increased nutrient and water uptake (Al Mahmud et al., 2019). Multiple studies have revealed that plant growth-promoting bacteria (PGPB) frequently release enzymes that breakdown the cell walls of phytopathogens, including proteases and chitinases (Chandra et al., 2018; Fadiji and Babalola, 2020; Sarkar et al., 2023). Furthermore, studies have shown that the synthesis of antibiotics such as phenazines, pyoluteorins, phloroglucinol, hydrogen cyanide, and pyrrolnitrin, along with siderophores and bacteriocins, can aid in inhibiting the growth of phytopathogens (Basu et al., 2022) as well as improving plant tolerance to various stresses (Abdelaal et al., 2021). Studies have previously suggested that introducing bacteria and other organisms that promote plant growth could enhance plant growth and health through additive or synergistic effects (Santoyo et al., 2021). For example, wheat plants inoculated with a biofilm-forming rhizospheric PGPB strain, i.e., *Pseudomonas azotoformans* FAP5, were able to ameliorate drought stress by increasing their photosynthetic potential and other physiological attributes (Ansari et al., 2021). Another study revealed that bacteria that can handle drought, such as *Bacillus subtilis* (LDR2), increase the resistance of wheat seedlings to drought stress by releasing phytohormones and extracellular polymeric substances (EPSs) (Barnawal et al., 2017). Ilyas et al. (2020) also reported that *Azospirillum brasilense* and *Bacillus subtilis* produced substantial amounts of EPS and osmolytes, which increased the ability of wheat plants to withstand drought. When these microbes were introduced at the same time, more ABA, proline, and EPS were produced. They also change the levels of phytohormones that are released during stress. Nevertheless, the introduction of these microorganisms (Ilyas et al., 2020) improved seed germination, plant growth, and the seedling vigor index in response to osmotic stress in wheat plants. Researchers have reported that increasing the activity of the jasmonic acid signaling pathway and reducing the synthesis of ethylene enhance drought tolerance in maize plants subjected to drought stress (Ansari and Ahmad, 2019; Chieb and Gachomo, 2023). In another study, rice plants inoculated with a PGPB strain from the rhizosphere i-e *Bacillus megaterium* PB50 presented greater amounts of chlorophyll, soluble proteins, and total soluble sugars than did plants that were not subjected to drought stress (Arun et al., 2020; Abd-El-Mageed et al., 2022; Hou et al., 2024). Additionally, these treated plants presented increased activity of peroxidase, superoxide dismutase, and catalase enzymes in response to drought stress in staple crops (Sandhya et al., 2010; Vardharajula et al., 2011; Batool et al., 2020; Joshi et al., 2020a; Joshi et al., 2020b; Ahmad et al., 2021a; Azeem et al., 2022).

### 5.5 Employing phytohormones in drought tolerance

Phytohormones play crucial roles in regulating different plant responses to diverse drought conditions, as shown in **Table 3**. Additionally, plant hormones such as ethylene, cytokinins, brassinosteroids, gibberellins, and auxin are very important for addressing drought stress (Ullah et al., 2018). Auxins, a group of phytohormones, have crucial functions in development, growth, and response to stress in plants (El Sabagh et al., 2022). The biosynthesis process for auxin synthesis, which mostly takes place in leaf primordia, immature leaves, and developing seeds, is mostly maintained by plant species. Phloem transport or cell-to-cell transfer are the means by which these substances are moved from their site of synthesis to the intended destination (Kou et al., 2022). Numerous studies by numerous researchers have demonstrated that auxin has a positive impact on the development of drought resistance. Aux/IAA genes have been found in rice, and studies have examined specific genes that are triggered in response to drought stress (Jung et al., 2015). Furthermore, drought stress has been linked to the Oryza sativa Indole-3-Acetic Acid Inducible 6 (OsIAA6) gene in rice. Another study by Mao et al. (2022) and Mega et al. (2023) examined wheat plants (TaPYLs) under drought stress and found that the overexpression of the Pyrabactin Resistance-Like gene resulted in the synthesis of excessive amounts of ABA, which strengthened the plants' resistance to drought by improving their water uptake and biomass characteristics. Another study of drought in wheat plants Onyemaobi et al. (2021) proposed that the role of the 9-cis-epoxycarotenoid dioxygenase (TaNCED) and ethylene response factor (TaERF) genes in the ABA and ethylene production pathways could regulate plant responses to environmental conditions (Magar et al., 2022). Additionally, auxin promotes root branching, which may be essential for increasing drought resistance (Roumeliotis et al., 2023). Other vital phytohormones known as cytokinins (CKs) are also important for controlling how plants grow, develop, and react to environmental stressors like drought (Ha et al., 2012). According to numerous studies, applying CK to maize, wheat, and rice plants under drought reduced the negative impacts of stress and promoted plant development (Raza et al., 2020; Wang et al., 2020b; Ostrowska et al., 2021; Iqbal et al., 2022; Kosakivska et al., 2022). According to several research, CKs can affect drought tolerance in both positive and negative ways. However, the length and intensity of drought can cause changes in CK levels. Increased endogenous CK levels were observed in transgenic plants that produced an isopentenyl transferase gene. By suppressing drought-induced leaf senescence and delaying senescence, the transgenic plants demonstrated enhanced drought tolerance. Several studies have indicated that CK negatively impacts drought tolerance in various plants (Prerostova et al., 2018). One well-known function of the CK oxidase/dehydrogenase (CKX) system is the degradation of CKs. The endogenous CK content in Arabidopsis is frequently decreased as a result of CKX overexpression (Nishiyama et al., 2011; Prerostova et al., 2018). Our understanding of the precise role of CK during drought stress and the particular channel by which it signals remains incomplete. Gibberellins (GAs) are very important plant hormones. They belong to a class of chemical molecules called tetracyclic diterpenoid carboxylic acids. In addition to acting as growth hormones, they can react to biotic and abiotic stressors. Applying GAs topically improves the drought resistance of maize, soybeans, and wheat, according to numerous research. These studies include (Akter et al., 2014; Nelissen et al., 2018; Wu et al., 2023a), soybean (Khan et al., 2022; Kang et al., 2014; Dong et al., 2019), and wheat (Plaza-Wüthrich et al., 2016; Yang et al., 2016; Al Mahmud et al., 2019).

### 5.6 Molecular approaches for enhancing drought tolerance in crops: transgenic and CRISPR/Cas9 innovations

Transgenesis and CRISPR/Cas9 gene editing are two molecular techniques that have greatly accelerated the development of drought-tolerant staple crops like wheat, maize, and rice. By precisely manipulating genes linked to stress, these technologies have improved agricultural performance in water-deficit situations and increased drought resilience. Significant gains in water shortage tolerance have been observed in rice when OsDREB1G and OsDREB2B, genes that are members of the DREBs (Drought Responsive Element Binding) family, are overexpressed. According to Chen et al. (2008), transgenic rice lines that expressed these genes showed survival rates of 83.27% and 85.57%, respectively, during drought circumstances (**Table 5**). Additionally, when rice plants were given the OsDREB1E gene, which is likewise implicated in the drought response system, they showed a moderate survival rate of 19.39% in the presence of water deficiency (Chen et al., 2008). According to Wang H. et al. (2022), CRISPR/Cas9-based editing of OsDREB1C, OsDREB1E, and OsDREB1G also improved resistance to heat, salt, and drought, indicating that CRISPR/Cas9 has the potential to produce rice types that are robust to several stresses. Transgenic expression of the OsWRKY76 gene, which controls jasmonate signaling via OsbHLH148 and provides enhanced drought tolerance, is another noteworthy example (Zhang et al., 2023).

**Table 5:** Transgenic and CRISPR/Cas9-modified crops exhibiting improved drought resistance.

Staple crop	Molecular Modification	Technique used	Drought tolerance Mechanism	Results obtained	Reference
Rice	OsDREB1G, OsDREB2B	Transgenesis	Enhanced tolerance to water scarcity	Survival rates of 83.27% for OsDREB1G and 85.57% for OsDREB2B under conditions of water deficiency	(Chen et al., 2008)
Rice	OsDREB1E	Transgenesis	Moderate water deficit tolerance	OsDREB1E survives 19.39% of water deficits.	(Chen et al., 2008)
Rice	OsDREB1C, OsDREB1E, OsDREB1G	CRISPR/Cas9	Improved drought tolerance	Increased drought, salt, and heat tolerance in transgenic rice.	(Wang H. et al., 2022)
Rice	OsWRKY76	Transgenesis	Improved drought tolerance	OsWRKY76 promotes drought tolerance in rice via OsbHLH148-mediated jasmonate signaling.	(Zhang et al., 2023)
Maize	ZmEREBP60	Transient transformation	Enhanced drought tolerance	Overexpressing ZmEREBP60 increased drought tolerance and reduced H2O2 and malondialdehyde buildup.	(Zhu et al., 2022)
Maize	ZmNHL2	Agrobacterium Transformation	Enhanced drought tolerance	ZmNHL2-overexpression transgenic lines have higher RWC and SOD/POD activity.	(Wang et al., 2024)
Maize	ZmMYB39	Agrobacterium Transformation	Overexpression of ZmMYB39 boosts maize drought resistance.	ZmMYB39 upregulates four downstream target genes (ZmRD22B, ZmDREB2A, ZmP5CS1, and ZmFNR1) to improve maize drought tolerance.	(Ren et al., 2024)
Maize/Arabidopsis	ZmLBD2	CRISPR/CAS9	Promote drought tolerance by increasing the growth attributes and antioxidants assays.	The ZmLBD2 gene improves plant H2O2 homeostasis and drought resistance.	(Jiao et al., 2022)
Wheat	TaDREB3	CRISPR/CAS9	Higher survival rates and higher yields	Enhanced the growth attributes	(Shavrukov et al., 2016)
Wheat	TaCBF5L	Agrobacterium tumefecians transformation	Improved wheat drought tolerance.	TaCBF5L expressed by the HDZI-4 promoter increased transgenic wheat grain yield significantly.	(Yang Y. et al., 2020)
Wheat	TaASR1-D	CRISPR/CAS9	ABA sensitivity and antioxidant capacity were increased in transgenic wheat plants by TaASR1-D.	More TaASR1-D was expressed in leaf vascular tissues and root and stem parenchyma cells.	(Qiu et al., 2021)

Similarly, the goal of genetic changes in maize has been to control oxidative stress and increase drought resistance. Drought tolerance was significantly improved by overexpressing ZmEREBP60, a gene that improves drought tolerance by reducing the buildup of reactive oxygen species such hydrogen peroxide (H2O2) and malondialdehyde (MDA) (Zhu et al., 2022). Similarly, by increasing relative water content (RWC) and antioxidant enzyme activities including superoxide dismutase (SOD) and peroxidase (POD), the ZmNHL2 gene, which was introduced via Agrobacterium-mediated transformation, improved drought tolerance (Wang et al., 2024). Additionally, the overexpression of ZmMYB39 in maize greatly enhanced drought tolerance by favorably regulating drought-responsive genes such as ZmRD22B, ZmDREB2A, ZmP5CS1, and ZmFNR1 (Ren et al., 2024). By controlling H2O2 homeostasis, a crucial component in sustaining plant growth under water stress, the CRISPR/Cas9-mediated editing of ZmLBD2 in maize and Arabidopsis further enhanced drought resistance (Jiao et al., 2022). To alleviate drought stress in wheat, a number of effective transgenic strategies have been used. When the TaDREB3 gene was altered with CRISPR/Cas9, it improved important growth characteristics and greatly increased survival rates and yields in drought circumstances (Shavrukov et al., 2016). Wheat is a great choice for breeding drought-tolerant wheat varieties because of the significant increase in grain production that resulted from the overexpression of TaCBF5L in wheat, which was triggered by the HDZI-4 promoter (Yang Y. et al., 2020). Another noteworthy instance is the CRISPR/Cas9 editing of TaASR1-D, which increased ABA sensitivity and antioxidant capacity while improving drought tolerance in transgenic wheat by modifying stress response pathways (Qiu et al., 2021). These molecular techniques, which range from precise genome editing using CRISPR/Cas9 to gene overexpression, show great potential in improving drought tolerance in a variety of staple crops. Along with increasing resilience in situations of water scarcity, these developments also provide light on the molecular processes behind how plants react to environmental stress.

## 6 Conclusion

Drought stress has a considerable negative impact on agricultural productivity worldwide because of protracted periods of below-average rainfall, which are frequently coupled with relatively high temperatures. The division of drought into meteorological, agricultural, and hydrological categories emphasizes the various barriers in terms of water accessibility which have an immediate influence on plant growth and productivity. Drought stress hampers plant metabolism, hinders physiological processes, and diminishes growth, resulting in significant yield reductions that can range from 30% to 90%, depending on the crop species and growth stage. Plants use adaptation and acclimation mechanisms to respond to drought stress. The physiological and biochemical makeup of plants changes quickly as they adapt to their new environment. As an illustration, the osmotic pressure is regulated, antioxidant mechanisms are activated, and their shape is evidently changed, with longer roots and smaller leaves. Long-term evolutionary changes, like the development of drought-resistant traits like xerophytism and succulence, are referred to as adaptation. Together, these processes enhance the plant's ability to maintain cellular integrity, control the water balance, and ensure successful reproduction in environments with limited water availability. Different modifications that improve plants' resistance to drought conditions have been the subject of recent investigations. Plant growth-promoting bacteria (PGPB) is an example of microbial inoculants that increase water and nutrient uptake during drought conditions; nanoparticles improve water absorption efficiency and stress resistance at the molecular level; and biochar applications increase soil water retention and nutrient availability. The integration of both traditional and novel approaches to drought mitigation and the creation of a comprehensive plan that integrates insights from molecular biology, biochemistry, and ecology to improve crop resilience to drought are noteworthy aspects of this review.

## 7 Future recommendations

1. In order to produce drought-tolerant crops, advanced genetic technologies should be used, emphasizing characteristics like deep root systems and increased water-use efficiency.
2. Plant growth-promoting microorganisms (PGPMs) require more study in order to improve water and nutrient uptake during drought stress, especially when employing novel microbial strains.
3. The usage of nanoparticles to increase stress resistance and water absorption while maintaining ecological safety needs more investigation.
4. To improve soil water retention and nutrient availability, along with organic amendments like biochar, its necessary to investigate other sustainable soil management strategies.
5. To improve water-use efficiency, drought-resilient crop rotations, precision-based irrigation methods, and climate forecasts are required.
6. To develop thorough models for drought management, it's critical to use an integrated systems approach that blends ecology, agronomy, and molecular biology.
